# Scaffolding the cup-shaped double membrane in autophagy

**DOI:** 10.1371/journal.pcbi.1005817

**Published:** 2017-10-24

**Authors:** Amir Houshang Bahrami, Mary G. Lin, Xuefeng Ren, James H. Hurley, Gerhard Hummer

**Affiliations:** 1 Department of Theoretical Biophysics, Max Planck Institute of Biophysics, Frankfurt am Main, Germany; 2 Department of Molecular and Cell Biology and California Institute for Quantitative Biosciences, University of California, Berkeley, California, United States of America; 3 Molecular Biophysics and Integrated Bioimaging Division, Lawrence Berkeley National Laboratory, Berkeley, California, United States of America; 4 Institute for Biophysics, Goethe University Frankfurt, Frankfurt am Main, Germany; University of Virginia, UNITED STATES

## Abstract

Autophagy is a physiological process for the recycling and degradation of cellular materials. Forming the autophagosome from the phagophore, a cup-shaped double-membrane vesicle, is a critical step in autophagy. The origin of the cup shape of the phagophore is poorly understood. In yeast, fusion of a small number of Atg9-containing vesicles is considered a key step in autophagosome biogenesis, aided by Atg1 complexes (ULK1 in mammals) localized at the preautophagosomal structure (PAS). In particular, the S-shaped Atg17-Atg31-Atg29 subcomplex of Atg1 is critical for phagophore nucleation at the PAS. To study this process, we simulated membrane remodeling processes in the presence and absence of membrane associated Atg17. We show that at least three vesicles need to fuse to induce the phagophore shape, consistent with experimental observations. However, fusion alone is not sufficient. Interactions with 34-nm long, S-shaped Atg17 complexes are required to overcome a substantial kinetic barrier in the transition to the cup-shaped phagophore. Our finding rationalizes the recruitment of Atg17 complexes to the yeast PAS, and their unusual shape. In control simulations without Atg17, with weakly binding Atg17, or with straight instead of S-shaped Atg17, the membrane shape transition did not occur. We confirm the critical role of Atg17-membrane interactions experimentally by showing that mutations of putative membrane interaction sites result in reduction or loss of autophagic activity in yeast. Fusion of a small number of vesicles followed by Atg17-guided membrane shape-remodeling thus emerges as a viable route to phagophore formation.

## Author summary

Cells use autophagy to recycle and degrade a wide range of materials, from individual molecules to entire organelles. We perform simulations to study a key step in the initial stages of the autophagy pathway: how the membrane adopts the characteristic cup shape of the phagophore that then engulfs its targets. We find that dimers of the protein Atg17 play a critical role in this membrane shape transition. Atg17 dimers form the 34-nm long, double-crescent shaped core of the Atg1 complex, which is known to be present at the “phagophore assembly site” of yeast. To study protein-assisted membrane remodeling, we integrate coarse-grained protein complexes into a model of membrane shape dynamics. In our simulations, we find that multiple Atg17 dimers cooperate to remove the barriers for the transitions from tube to disk shapes, and from disk to phagophore shapes. In experiment, we confirmed that perturbations of Atg17 membrane interaction have a substantial influence on autophagy induction, up to a to complete loss of detectable autophagic activity. Our combination of simulations and experiments thus shed light on a membrane-shaping function of the unusually shaped Atg1 complex in phagophore assembly.

## Introduction

Autophagy, a physiological process used by eukaryotic cells to degrade cytoplasmic materials, is initiated under stress conditions such as nutrient starvation [1–4]. Among other processes, autophagy is associated with cancer, cellular aging, the immune system, and infection [5–9].

Macroautophagy, one of various autophagy types, proceeds as a sequence of membrane remodeling events, ultimately leading to the engulfment and degradation of cytosolic components. Autophagosome biogenesis is accomplished by the formation of the phagophore (also called the isolation membrane) at the preautophagosomal structure/phagophore assembly-site (PAS). The formation of the phagophore and its dynamic transition from the initial membrane source are not well understood. For yeast, it is thought that the fusion of approximately three Atg9-containing vesicles [10] (in the following denoted as Atg9 vesicles), originating from the Golgi, initiates phagophore formation [11, 12]. The subsequent expansion of the membrane is thought to derive from multiple membrane sources, with the endoplasmic reticulum (ER) most prominent among them [13].

The energetic cost of membrane deformation is large on a thermal scale. On any pathway towards the cup-shaped phagophore, one thus needs to consider the possibly substantial energy barriers imposed by membrane elasticity [14]. Knorr et al. [15] recently explored the transition from disk shapes to cup shapes. Whereas the corresponding barrier disappears beyond a critical disk diameter-to-thickness ratio, it is not clear how disk-shaped double membrane structures form if phagophores are initiated by vesicle fusion.

Here, we examine the energetics of phagophore initiation, starting from a point immediately after Atg9 vesicle fusion [16, 17]. In addition to the disk-to-cup barrier, we find an even larger barrier that prevents the transition to disk shapes, starting from initial fused vesicles. However, evidence abounds that protein complexes play a critical role in the phagophore membrane remodeling process [18–20]. Indeed, we show that the cooperative action of the S-shaped Atg17 complexes [21] localized at the PAS [22, 23] help overcome both barriers, even for symmetric membranes with negligible spontaneous curvature.

To study protein-aided phagophore formation, and membrane remodeling more generally, we develop a simulation model that adds explicit membrane-interacting proteins to the Helfrich [24] membrane elastic description. We represent the membrane as a dynamically triangulated mesh [25–27]. In this way, we can simulate the coupled dynamics of the membrane shape and of membrane-associated protein complexes. By varying the binding strength of the Atg17 complex and its shape, we identify the factors required to transform tubular membrane structures formed immediately after fusion into the cup-shaped phagophores that ultimately lead to the autophagosome. Membrane triangulated models have been widely used to study membrane fluctuations and shape transformations [25, 28–31] and the interactions between membranes and nanoparticles [26, 27].

## Results

To set the stage, we first review how the preferred shape of a membrane vesicle depends on its reduced volume $v=6\sqrt{\pi }V/A^{3/2}$ [32, 33], where *V* is its volume and *A* its area. As *v* is lowered, starting from a spherical shape (*v* = 1), free vesicles undergo two transitions between three globally stable axisymmetric shapes, from prolate ellipsoids (0.652 ⩽ *v* ⩽ 1) over biconcave oblate ellipsoids (0.592 ⩽ *v* < 0.652) to cup shapes (0 < *v* < 0.592). The first transition is discontinuous, whereas the second one is continuous [32, 33]. The prolate and oblate ellipsoidal shapes resemble elongated tubular vesicles (tubes) and biconcave shapes (disks), respectively. Phagophores correspond to cup shapes, with their characteristic double-membrane structure. Hereafter we simply refer to these three locally stable conformations as tube, disk, and phagophore or cup.

**Fig 1:**
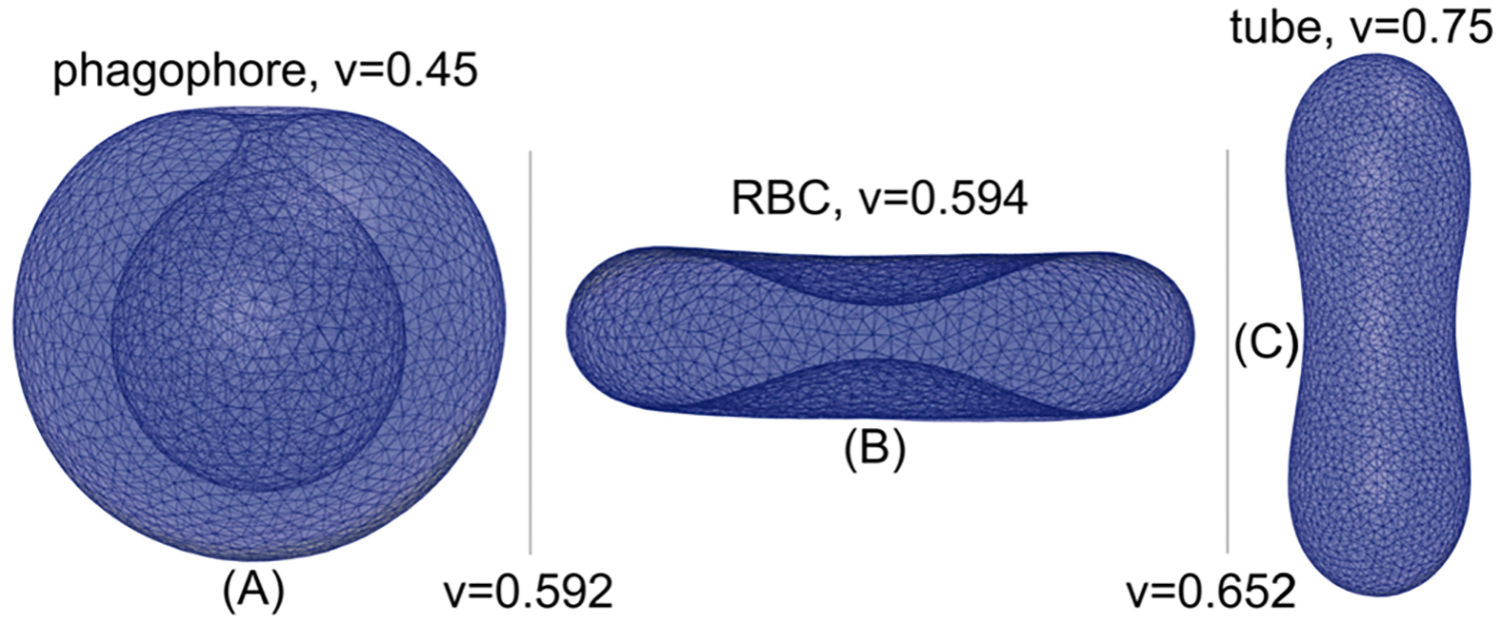
Stable vesicle shapes for different reduced volumes. **(A)** Axisymmetric cup-shaped vesicle (phagophore) with reduced volume *v* = 0.45. **(B)** Disk-shaped (axisymmetric biconcave oblate) vesicle with reduced volume *v* = 0.594. **(C)** Axisymmetric prolate vesicle (tube) with reduced volume *v* = 0.75. The transitions occur at reduced volumes *v* ≈ 0.652 and *v* ≈ 0.592 from tube to disk, and from disk to phagophore, respectively [32, 33]. The energetics of the membrane tube-to-sheet transition in the regime of narrow tubes is explored in more detail in [14].

Fig 1 shows stable tube, disk, and phagophore vesicle shapes corresponding to reduced volumes *v* = 0.75, 0.594, and 0.45. Vertical lines indicate the two transitions from tube to disk and disk to phagophore that occur at reduced volumes *v* ≈ 0.652 and *v* ≈ 0.592, respectively. The discontinuous transition between the tube and the disk occurs through intermediate nonaxisymmetric vesicles. The second transition between the disk and the phagophore, however, occurs through intermediate axisymmetric vesicles.

Area- and volume-conserving fusion of equal-size vesicles lowers the reduced volume. According to Eq (2), the reduced volumes of *n* = 2, 3, and 4 fused vesicles are *v*_2_ = 0.707, *v*_3_ = 0.577, and *v*_4_ = 0.5, respectively. Comparing these reduced volumes to the shape diagram in Fig 1, we find that already for *n* = 3 fused vesicles, the phagophore is the overall preferred shape, because *v*_3_ = 0.577 < 0.592. For fusion of vesicles of unequal size, the reduced volume will be higher. As our first major result, we therefore conclude that fusion of at least three vesicles is required to induce the phagophore shape, lacking other stabilizing factors.

However, membrane elasticity theory also implies that the transition towards the phagophore shape is not straightforward [14]. The post-fusion vesicle has to cross over two energy barriers to reach the phagophore structure [32, 33]. These barriers correspond to a discontinuous transition from tube to disk and a continuous transition from disk to phagophore. The small margin between *v*_3_ = 0.577 and the second transition volume *v* = 0.592 exacerbates the problem. Over finite ranges of the reduced volume, co-existence between stable and metastable shapes result in hysteresis effect. The transitions and the subsequent phagophore formation are thus far from trivial.

In the following, we investigate the membrane remodeling of a vesicle with a reduced volume *v* = *v*_3_ = 0.577, starting from initial fused configurations of three vesicles in linear arrangement. As we will show first for free vesicles, in the absence of proteins, this remodeling confronts two energy barriers (or one if *n* ⩾ 4 vesicles fused). In a second step, we then try to identify the effect of Atg17 protein complexes on the barrier energies and the transition process.

In our simulations with and without Atg17 complexes, the vesicle volume is preserved. The volume of vesicles permeable to water but not osmolytes is controlled by the osmolarity difference between the vesicle interior and exterior. Any water flux into or out of an otherwise impermeable vesicle builds up a counteracting gradient in water activity. Therefore, the vesicle internal volume remains nearly constant during the transition towards the phagophore even in conditions where a volume expansion would be favored by membrane elastic energy.

### Free vesicle

At a fixed reduced volume *v* = 0.577 of the vesicle, both tube-to-disk and disk-to-phagophore transitions take place through intermediate nonaxisymmetric and axisymmetric shapes, respectively. We can characterize these intermediate shapes by varying the area difference Δ*a* between the bilayer leaflets. The area difference acts as a reaction coordinate that determines the various shapes of the vesicle at a given reduced volume *v* [34, 35]. We performed MC and SA simulations of a triangulated vesicle based on the Helfrich model. As starting point, we used a fused vesicle, described as a single simply-connected topological surface. To represent the moment immediately after the fusion of the three smaller vesicles, the initial shape matches three spheres punctured at their contact points and connected by narrow membrane catenoid necks.

**Fig 2:**
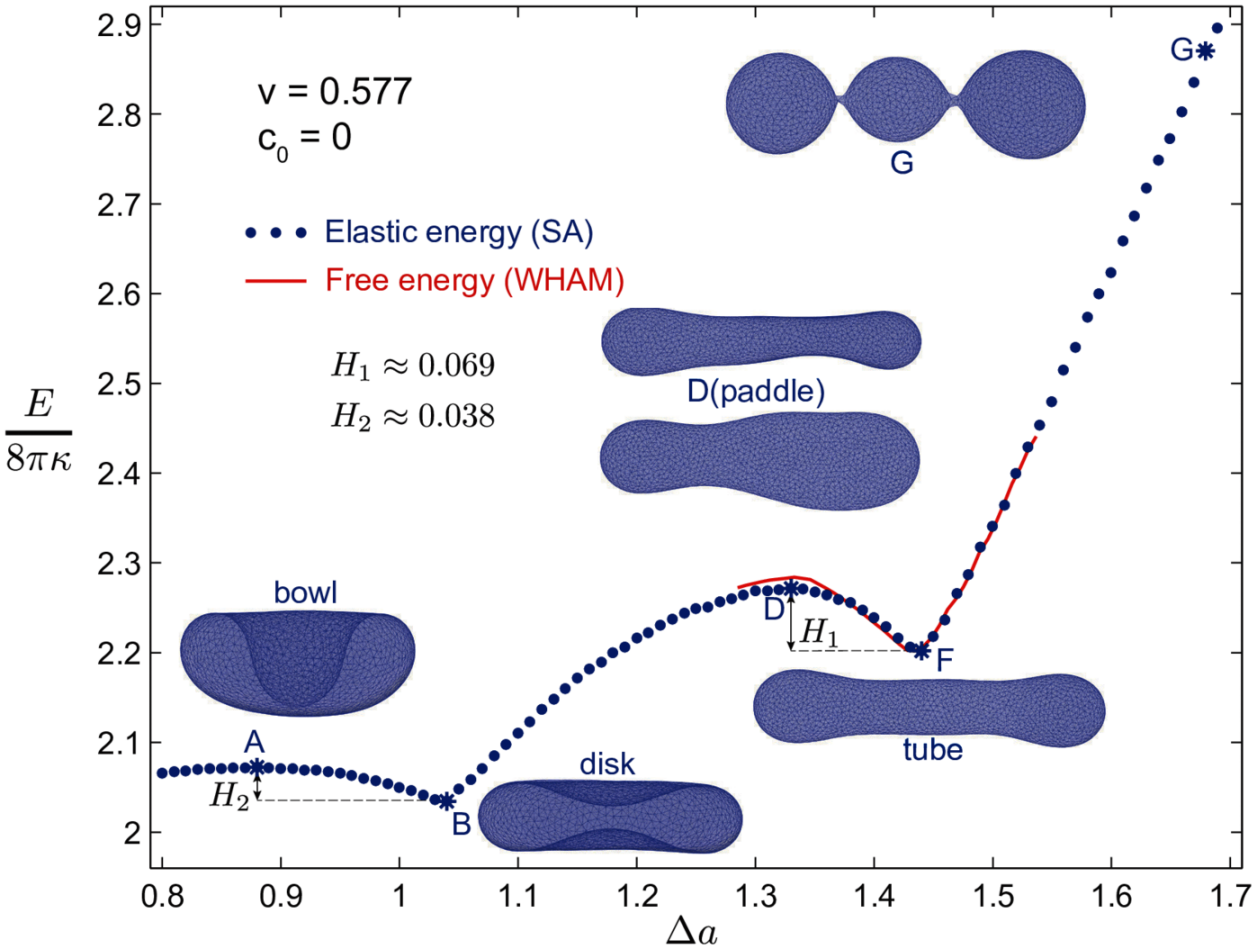
Membrane energy of different vesicle shapes at reduced volume *v* = *v*_3_ = 0.577. The elastic energy (blue dots) was determined by SA simulations as a function of the area differences Δ*a*. Energies are reported in units of 8*πκ*, which is about 8*πκ* ≈ 250 to 500 *k*_B_*T* for typical membrane rigidities of *κ* ≈ 10 to 20 *k*_B_*T*. Arrows indicate the two energy barriers *H*_1_ ≈ 0.069(8*πκ*) and *H*_2_ ≈ 0.038(8*πκ*) for the tube-to-disk and disk-to-phagophore transitions, respectively. Also shown are characteristic membrane shapes (A) at the top of the barrier between disk and phagophore, (B) in the metastable disk minimum, (D) at the top of the tube-to-disk barrier, (F) in the metastable tube minimum, and (G) of three just-fused spheres for a large area difference. The free energy (red), obtained by combining umbrella sampling simulations using WHAM, indicates a slightly higher free energy barrier from entropic effects. See [14] for a more extensive exploration of the tube-to-sheet transition in membrane vesicles.

To map out the shape transformation, we performed SA simulations of a triangulated vesicle at a constant reduced volume of *v* = *v*_3_ = 0.577 and different area differences Δ*a*. The rescaled bending energy *E*/(8*πκ*) plotted as a function of Δ*a* in Fig 2 shows two energy barriers *H*_1_ ≈ 0.069 (8*πκ*) between the tube and disk conformations, and *H*_2_ ≈ 0.038 (8*πκ*) between the disk and phagophore conformations. Starting with high Δ*a* and moving down the shape branch, the vesicle starts from three connected spheres in a linear arrangement at Δ*a* > 1.65 and forms a metastable tubular shape (Δ*a* ≈ 1.44; point F). A further reduction of Δ*a* carries the vesicle over a barrier by inducing a paddle shape (Δ*a* ≈ 1.33; point D). From this point on, the vesicle moves downhill on the energy curve. The wide side of the paddle grows and the narrow side shrinks to join the wide side, eventually forming a metastable disk shape (Δ*a* = 1.04; point B). The transition from the disk to the phagophore occurs over the second barrier *H*_2_, with a bowl-shaped structure at the barrier top (point A). This second energy barrier of the shape branch, *H*_2_, vanishes for lower reduced volumes *v* < 0.52 [35], a regime not quite reached with three fused vesicles, *v*_3_ = 0.577, but already with four, *v*_4_ = 0.5. However, already for three fused spheres the phagophore shape constitutes the global minimum, located to the left of point A.

The high energy barrier *H*_1_ for three fused vesicles at *v* = *v*_3_ delays or even prevents the tube-to-disk transition under normal condition in the absence of any additional driving mechanism. For typical membrane bending rigidities of *κ* = 10 to 20 *k*_B_*T*, the energy barrier *H*_1_ ≈ 17 to 34 *k*_B_*T* is high on a thermal scale [29, 35]. One might expect that the transition from tube to disk shapes might be aided by fusing more vesicles to the tubular vesicle at *v* = *v*_3_, which lowers the reduced volume $v=1/\sqrt{n}$ and thus increases the driving force. To investigate the role of the number of fused vesicles on the barrier height and the tube-to-disk transition, we performed SA simulations at different reduced volumes $v=1/\sqrt{n}=0.5$, 0.447, 0.4, 0.378 corresponding to *n* = 4 to 7 fused vesicles. The rescaled bending energies of the shape branch for these reduced volumes are shown in S1 Fig. See also [14]. Interestingly, we find that the energy barrier does not shrink when the reduced volume decreases by fusing more vesicles. As shown in S1 Fig the energy barrier is approximately identical *H*_1_ ≈ 0.07 (8*πκ*) for different numbers of fused vesicles *n* = 4 to 7. Consequently if the transition to the phagophore does not occur for three fused vesicles, adding more vesicles does not speed it up. If a barrier of 17 to 34 *k*_B_*T* for a spontaneous disk-to-phagophore transition is indeed insurmountable on relevant timescales, another mechanism has to carry the vesicle over the barriers for phagophore formation by vesicle fusion to be a viable pathway.

By calculating free energy profiles that include entropic contributions, we ensured that the tubular membrane structures obtained from fusion of three vesicles are metastable also in the presence of thermal fluctuations of the membrane shape. Starting from initial vesicle structures around the energy barrier obtained from SA simulations, we performed umbrella sampling simulations by applying biasing harmonic potentials on the area difference as our reaction coordinate. The results were then combined using the Weighted Histogram Analysis Method (WHAM) [36] to determine the potential of mean force, i.e., the free energy profile as a function of Δ*a*. The red curve in Fig 2 shows a slightly higher energy barrier in the free energy curve compared to the SA simulation results. The free energy barrier amounts to 21 to 42 *k*_B_*T* for bending rigidities *κ* = 10 to 20 *k*_B_*T*, respectively.

As a further test of the metastability of tubular structures beyond the Helfrich model, we performed molecular dynamics simulations of three newly fused vesicles. Our vesicle triangulated model has been shown to be capable of accurate Helfrich bending energy calculations even for sharply curved vesicles interacting with nanoparticles [26, 27]. The Helfrich model, however, is only valid when the membrane thickness is negligible compared to the other membrane dimensions such as vesicle radius. Moreover, by using the Helfrich model we ignore asymmetry in the membrane leaflets. Atg9 vesicles with a typical size of 20 to 50 *nm* in diameter [10, 11], are indeed asymmetric in terms of the number of lipids in their two leaflets. Moreover, the typical membrane thickness of 4 to 5 *nm* is not negligible compared to the vesicle radius. Therefore, to ensure the stability of the tubular structure and the spontaneous transition from the pearled post-fusion structure towards the tubular morphology, we performed coarse-grained simulations of three newly fused vesicles using the MARTINI model [37] to follow the subsequent shape relaxation.

**Fig 3:**
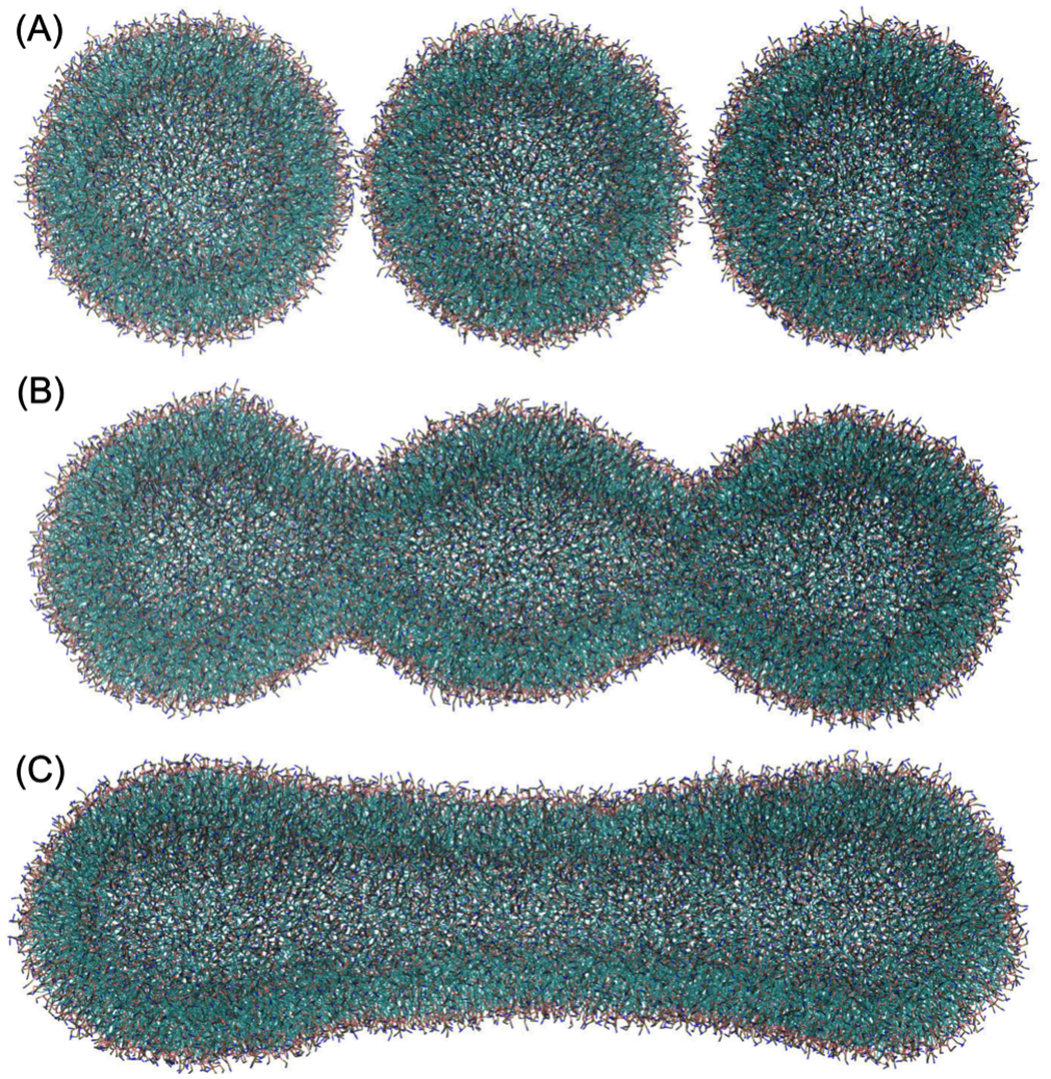
Coarse-grained MARTINI simulation of three fused vesicles. (A) Three initial POPC vesicles each composed of 2048 POPC lipids just before fusion. (B) Post fusion structures of three vesicles fused together by gradually pulling lipids together from contacting vesicles to prevent water exchange between the inside and the outside of the vesicles. (C) Equilibrated tubular structure after 0.8 *μ*s simulation. The post fusion structure composed of 6144 POPC lipids rapidly transitioned to a tubular vesicle which is stable during 1.2*μ*s (see S1 Video). The 1.1 × 10^6^ water particles are not shown.

The vesicle size was chosen to be 20 *nm* in diameter, as the most critical Atg9 vesicle diameter at the low end of the experimental range [10, 11]. The vesicles were first fused by pulling neighbor groups of lipids together, with water exchange prevented during the fusion process. The post-fusion structure was then equilibrated using molecular dynamics simulations. An initially pearled vesicle transformed into a tubular structure that remained stable for the entire, rather long simulation of 1.2 *μ*s, as shown in S1 Video. The initially pearled tube, as obtained after fusion, gradually straightened out to form a tube of near constant diameter (see Fig 3). During the 1.2 *μ*s long simulation, the overall tubular structure persisted, which is consistent with the metastability expected on the basis of the membrane elastic model.

We also combined Kramers theory with molecular dynamics simulations to assert the kinetic stability of the tubular shape. From the MARTINI molecular dynamics simulations, we estimated a characteristic time of vesicle shape fluctuations using the vesicle radius of gyration *R*_*g*_ as observable. After a fast “molecular” relaxation, the autocorrelation function of *R*_*g*_ exhibits a slow phase with a time constant of *τ* ≈ 0.05 *μ*s (S2 Fig). Within a Kramers approximation, the escape time from the tubular state is 2*πτ* exp(*H*_1_/*k*_B_*T*), or about 7 min to ≫1 day for the estimated free energy barriers *H*_1_ between 21 and 42 *k*_B_*T*. The barrier *H*_1_ is thus effectively insurmountable on relevant timescales.

### Atg17 dimers induce phagophore shape

The Atg1/ULK1 complex is a possible candidate to mediate the vesicle shape transformation required to induce the phagophore shape. Atg9 vesicles are believed to be tethered together by the Atg1-kinase complex, with the Atg13 HORMA domain [38] binding to the N-terminus of Atg9 [39]. Atg17 dimerization is central to the organization of the Atg1 complex, and the dimer interface is critical for function in autophagy [22]. On the basis of quantitative fluorescence microscopy, between 7 and 28 dimers of Atg17 localize to the PAS [23, 40]. In crystals and in solution, the Atg17 dimer adopts a unique, S-shaped double crescent [22, 23, 41]. The concave surface of the crescent is interrupted by the Atg29-Atg31 subcomplex [22]; however, the subcomplex is mobile and it is thought that it could become displaced upon activation [42]. The Atg17 crescent shape appears to be ideally suited to interact with highly curved membrane surfaces of the dimension of the phagophore initiating vesicles and/or phagophore rim. The detailed role and mechanism of Atg17 in phagophore initiation has been exceptionally difficult to probe experimentally, and is therefore a highly appropriate target for in silico investigation.

**Fig 4:**
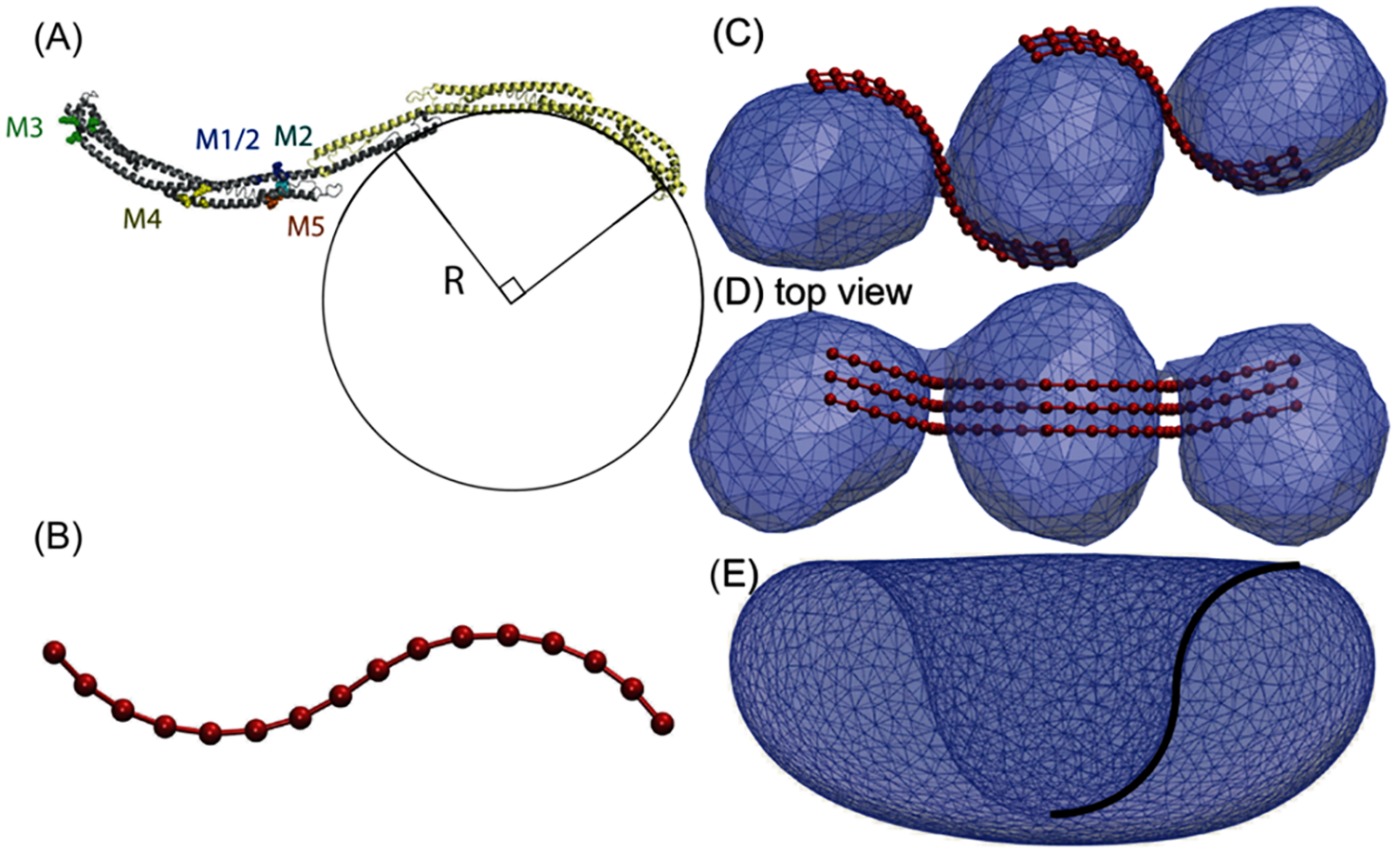
Atg17 dimer structure and membrane interaction. (A) Atg17 dimer in ribbon representation. The circle indicates the crescent curvature. Mutant clusters in one of the two copies of Atg17 are indicated with a space-filling atomic model. M1, blue; M2 unique residues, cyan; M3, green; M4, yellow; and M5 orange. (B) Coarse-grained representation of Atg17 dimer. (C) Side and (D) top views of three just-fused vesicles tethered by two groups of three Atg17 dimers. The snapshot shows the very first moment after fusion when two narrow catenoid membranes connect the vesicle pairs. (E) The Atg17 dimer matches the profile of the bowl membrane at the top of the second barrier (see point A in Fig 2) between disk and phagophore conformations.

In the following, we will probe a possible role of the Atg1 complex in phagophore shape induction by performing simulations of membrane shape transformations in the presence of membrane-adhered Atg17 complexes. Fig 4A shows the structure of the Atg17 scaffold of the Atg1 complex based on the crystal structure (PDB code: 4HPQ) [22]. The Atg17 dimer has multiple basic patches and protruding hydrophobic residues on its surface that indicate possible membrane interaction sites (Fig 4A). Below, we report experiments probing these interactions. To represent the spread-out interaction surface in our coarse-grained model (see Materials and methods), all beads interact uniformly with the membrane.

The simulations start from a configuration immediately after fusion of three Atg9 vesicles (Fig 4C and 4D). Following the suggestions in Refs [11, 22, 23, 42], the three fused Atg9 vesicles are held together by Atg17 dimers. Atg17 dimers are arranged to tether the fused vesicles together, as proposed in recent models [11, 22, 42]. Without loss of generality, because of rotational symmetry of the dimers around the fusion axis, the Atg17 dimers are initially located on one side of the connecting necks of the fused vesicles. Fig 4C and 4D illustrates the post-fusion structure of three vesicles with six Atg17 dimers in two groups responsible for tethering pairs of vesicles. Starting from this configuration, we performed simulations in which the membrane shape and the adhered Atg17 dimers evolve dynamically on their coupled energy surfaces, with preserved reduced volume *v* = *v*_3_ = 0.577 and vesicle area.

### Intermediate binding: The paddle membrane pathway

**Fig 5:**
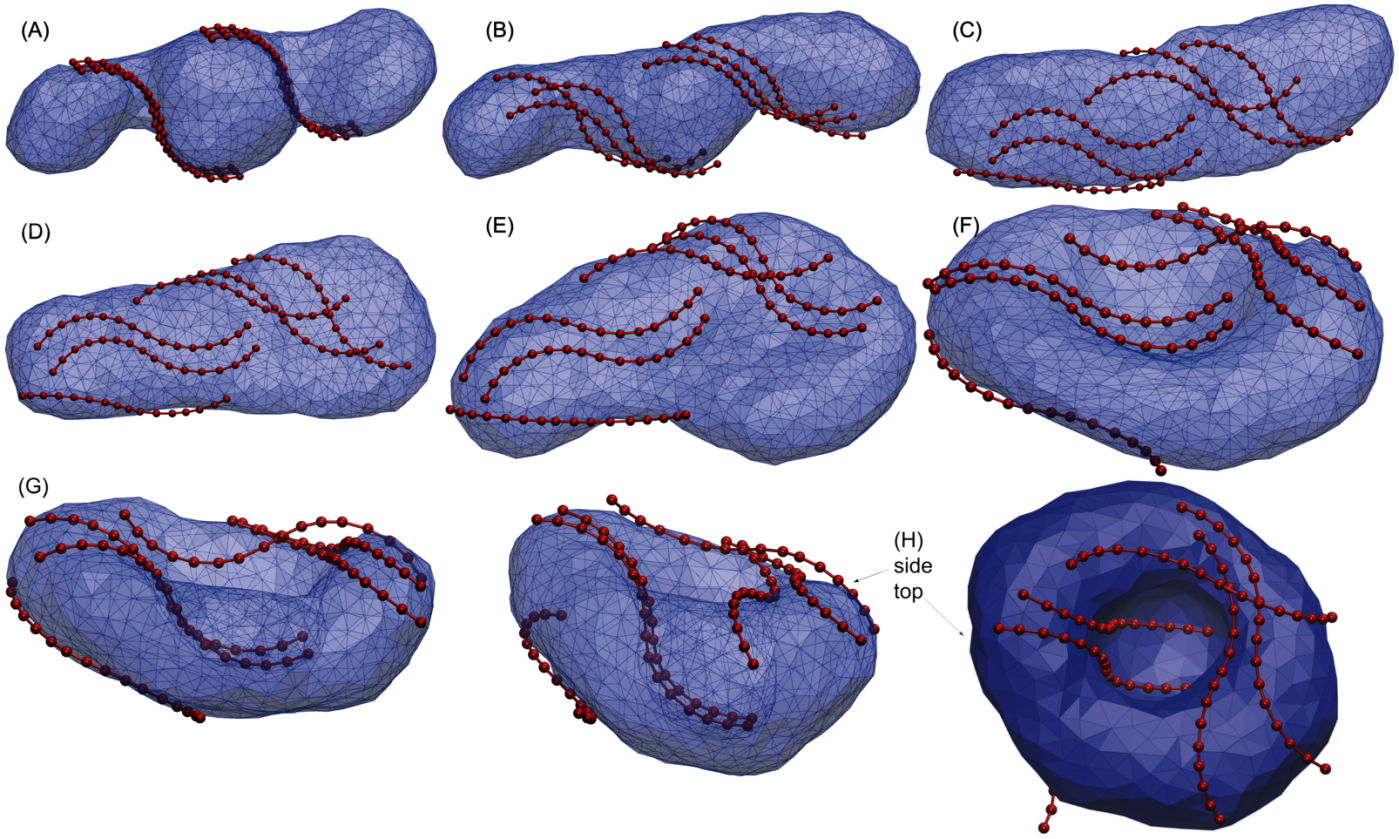
Phagophore induction by Atg17 dimers in the intermediate binding regime: The paddle pathway. (A-H) Snapshots taken along a simulation of Atg17-induced phagophore induction with an Atg17-membrane binding strength of *u* = 0.12 (membrane: transparent blue; Atg17 dimers: red). (A) Vesicle after fusion (with *v* = *v*_3_ = 0.577) and opening of the two catenoid shaped necks, interacting with six Atg17 dimers. The initial vesicle transforms to the bowl (F,G) and the phagophore (H) through intermediate paddle (D) conformations. (H) Phagophore shape shown in transparent side view (left) and opaque top view (right).

We found that Atg17 binding of sufficient strength (*u* > 0.07) was indeed able to induce spontaneous transitions to the phagophore shape. First, the narrow catenoid necks between the fused Atg9 vesicles opened (Fig 5A). Then, in an intermediate regime of binding strengths (0.07 < *u* < 0.14), the vesicles transformed into a paddle structure. From there, in a sequence of membrane motions and protein rearrangements, the phagophore cup shape was formed (Fig 5H).

Fig 5 illustrates the sequence of membrane conformations of the paddle pathway obtained with binding strength *u* = 0.12, which is typical of the intermediate regime (0.07 < *u* < 0.14). S2 Video shows an Atg17-mediated phagophore induction event in this regime. The paddle conformation is induced by three Atg17 dimers on the right side of the vesicle in Fig 5C–5E. The shape of Atg17 dimers helps in escaping the metastable tubular conformation and crossing over the first energy barrier *H*_1_. Atg17 binding stabilizes the paddle conformation and prevents it from going back to the tube. The vesicle widens at one end, resulting in an ellipsoidal cross section somewhere along the tube. The ellipsoidal section of the paddle then grows and absorbs the paddle tail. In the trajectory shown, this widening is aided by two parallel dimers observed on the left side of Fig 5D–5F, which stabilize the concave side of the membrane with their crescents and jointly help to cross the second barrier between the disk and phagophore. The S-shaped Atg17 dimers simultaneously bind to the membrane on the convex side of its first half and the concave side of the second half. While the concave side of the first half binds to the convex edge of the cup, the convex side of the other half forms the concave inner side of the cup. Cooperative arrangement of a few or several dimers thus forms concave-flat bowl shapes, as seen in Fig 5F and 5G. The transition from the concave-flat bowl at the top of the barrier *H*_2_ (see point A in Fig 2) towards the convex-concave phagophore then occurs spontaneously.

### Strong binding: The starfish pathway

**Fig 6:**
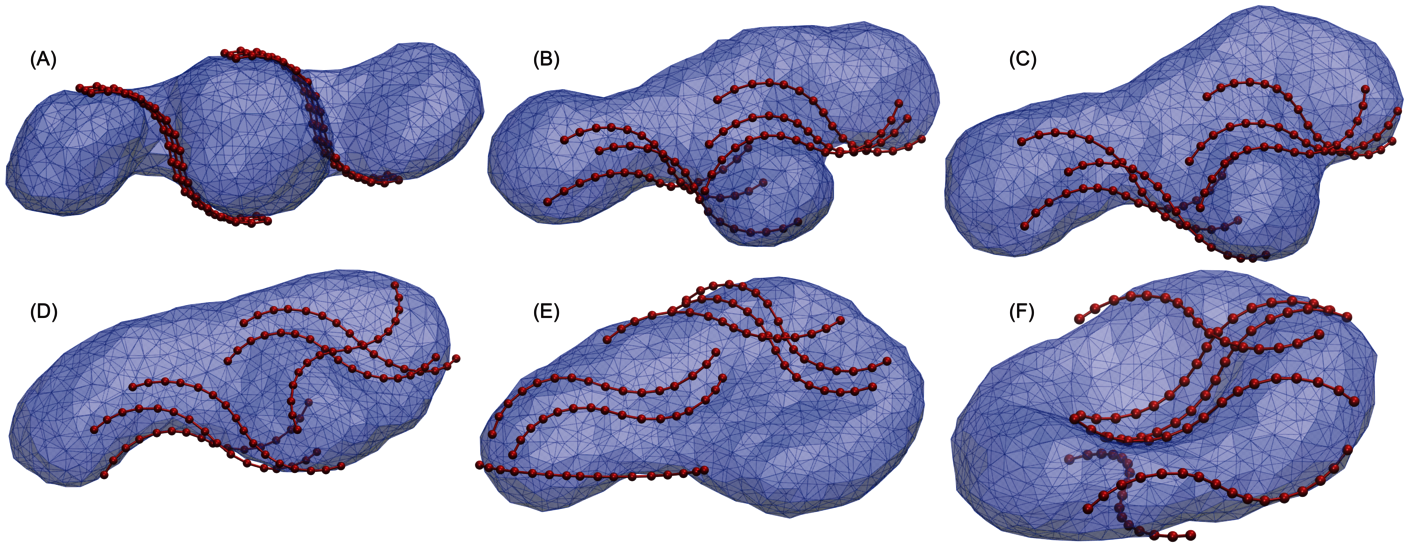
Strong binding regime: The starfish pathway. (A-F) Snapshots taken along a simulation of Atg17-induced phagophore induction with an Atg17-membrane binding strength of *u* = 0.17 (membrane: transparent blue; Atg17 dimers: red). The initial vesicle (A) with *v* = *v*_3_ = 0.577 transforms to the bowl (F) through intermediate starfish conformations (B,C), induced by strong lateral binding of Atg17 dimers (three right strands in (D,E)) and their cooperative interactions. The transitions from bowl toward the phagophore is similar to that in the paddle pathway of Fig 5.

Also for strong binding, *u* > 0.14, Atg17 dimers are able to induce a transition to the phagophore cup shape. However, the transition pathway is different from that in the intermediate regime. Fig 6 shows snapshots from a simulation with *u* = 0.17. The strong and cooperative interactions of Atg17 dimers with the membrane stabilize a hemispherical protrusion at the center of the vesicle (Fig 6B). Its neck is embraced by dimer crescents on opposite sides. The remaining membrane, originating from the two vesicles at the ends, interacts only weakly with Atg17 dimers. Deformation of this uncoated membrane causes widening (Fig 6B) and the adoption of a starfish-like conformation (Fig 6C). The starfish shape is, however, unstable due to its high bending energy and deforms into a paddle shape by losing binding interactions with some dimers (Fig 6D). The final steps follow the paddle pathway, with a paddle-like shape transitioning into a bowl conformation (Fig 6E and 6F). S3 Video shows an Atg17-mediated phagophore induction event along the starfish pathway, up to the point where the bowl shape is reached. The subsequent transition to the cup shape proceeds as in S2 Video.

### Weak binding regime: The tubular trap

**Fig 7:**
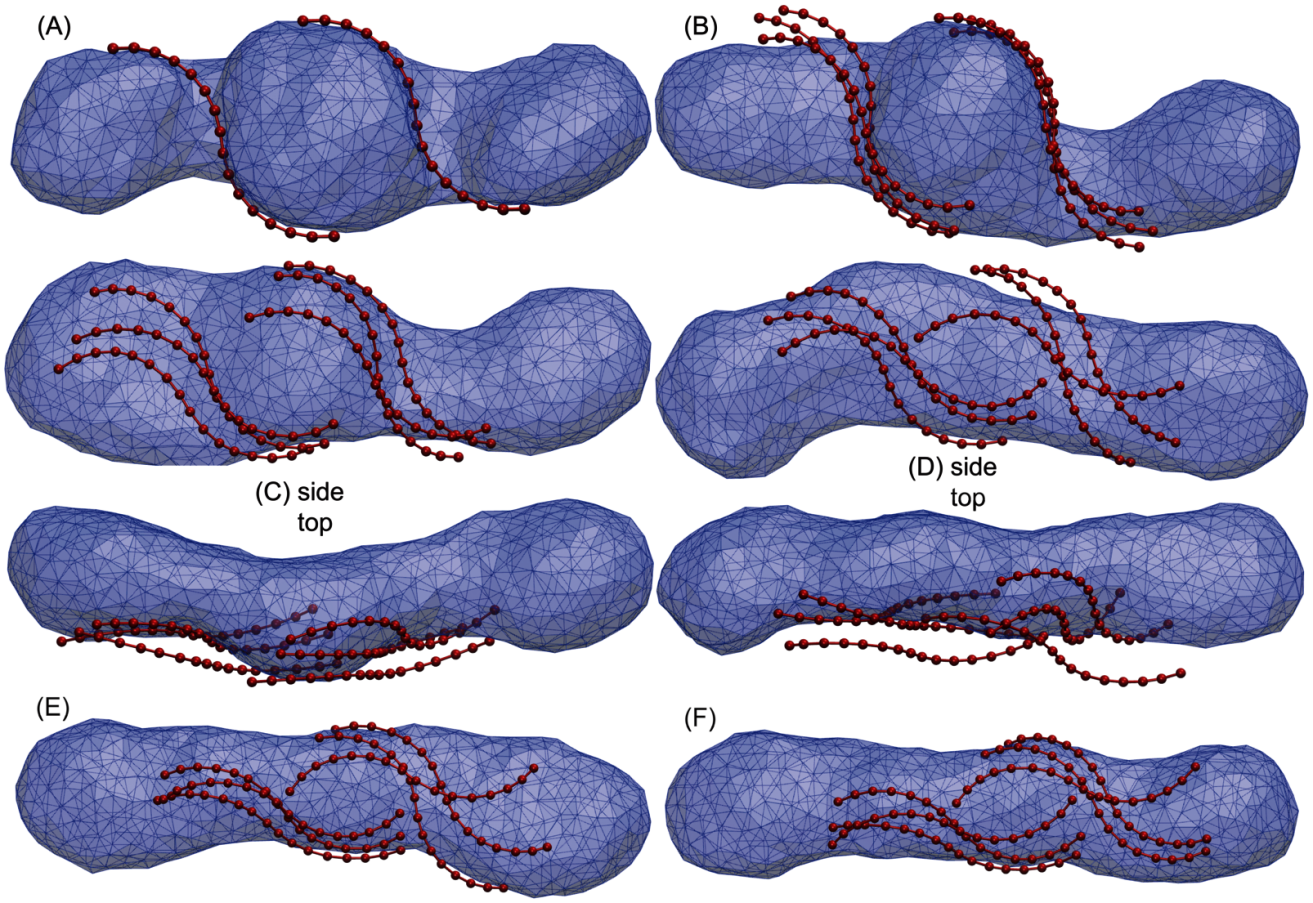
Tubular trap in the weak binding regime. (A-F) cooperative binding of six single-stranded Atg17 dimers in two groups of three. Although a paddle-like shape starts to form in snapshot C, the weak binding strength *u* = 0.05 of the dimers, is not strong enough to stabilize the paddle. Eventually the vesicle moves back to the unfavorable tube shape (D-F) and gets trapped there.

In the weak binding regime with binding strengths *u* ⩽ 0.07, the membrane remodeling is barely influenced by Atg17 dimers. As shown in Fig 7B, the membrane releases its high bending energy by partial separation from Atg17 dimers. Fig 7 illustrates the sequence of membrane structures for a binding strength *u* = 0.05. Partially unbound dimers are observed on the upper section of the left dimer group in Fig 7B. The dimers almost completely unbind as shown in the top views (C) and (D) of the intermediate conformations. The weak binding of the dimers to the membrane is not strong enough to prevent membrane transformation into the tubular structure.

During membrane remodeling, intermediate paddle-like structures occasionally form by the cooperative interactions between two or more dimers, as seen on the left side of Fig 7C. However, the weak binding and thermal fluctuations induce dimer unbinding and transform the unsustainable paddle shapes back to the tubular structure. Eventually the vesicle adopts a tubular shape, as seen in Fig 7D–7F, and gets trapped in front of the barrier *H*_1_ of Fig 2. Although the cooperative interactions of the dimers tend to induce paddle formations, the paddle does not persist due to weak membrane binding. We conclude that in the weak binding regime, a high energy barrier prevents crossing from the tube to the disk, in effect trapping the fused vesicle in a tubular conformation.

### Straight protein chains

**Fig 8:**
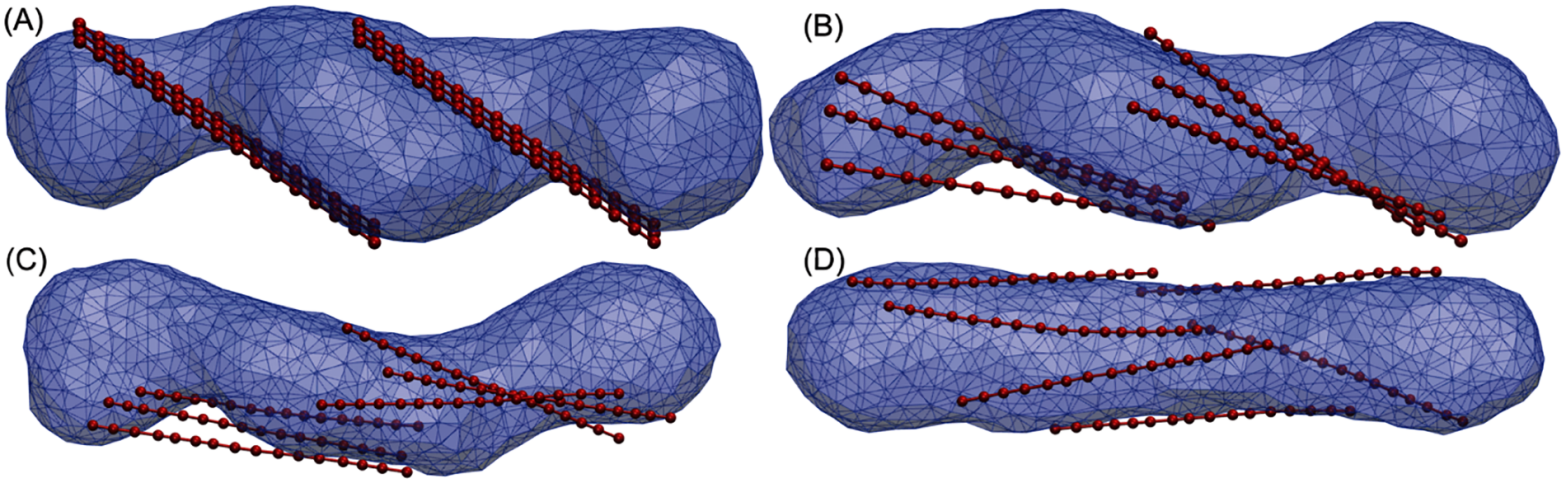
Membrane remodeling with straight proteins. (A-D) Snapshots taken along a simulation of attempted phagophore induction with a straight variant of an Atg17-like protein and a membrane binding strength of *u* = 0.1 (membrane: transparent blue; Atg17 dimers: red).

Our simulations highlight the significant role of the unique S-shape of Atg17 complexes in the phagophore induction process. We found that the double-crescent shape of the Atg17 dimers aids in the formation of the double-membrane phagophore conformation. To highlight the role of the dimer shape, we performed simulations with six strands of straight proteins. Similar to our previous simulations, the six strands are initially arranged on the three-vesicle structure after the fusion process. Fig 8 shows a few snapshots of membrane remodeling with straight protein chains in the intermediate adhesion regime. Straight protein chains fail to induce the phagophore structure and instead transform the membrane into an unfavorable tubular conformation. This emphasizes the crucial role of a relatively rigid crescent shape of the Atg17 dimers in transforming the membrane towards the cup-shaped phagophore shape.

### Atg17 dimer flexibility and membrane properties

In our model, we assumed very stiff Atg17 dimers by creating rather high bending moments acting on the bending angles of the dimer chains. However, real Atg17 dimers might experience some flexibility of their double-crescent shapes. To understand the role of Atg17 flexibility, we performed simulations in a typical intermediate binding regime with *u* = 0.1 and for several values of the Atg17 bending stiffness (see Materials and methods) with six Atg17 dimers on three fused vesicles with identical initial structures as in Fig 5.

**Fig 9:**
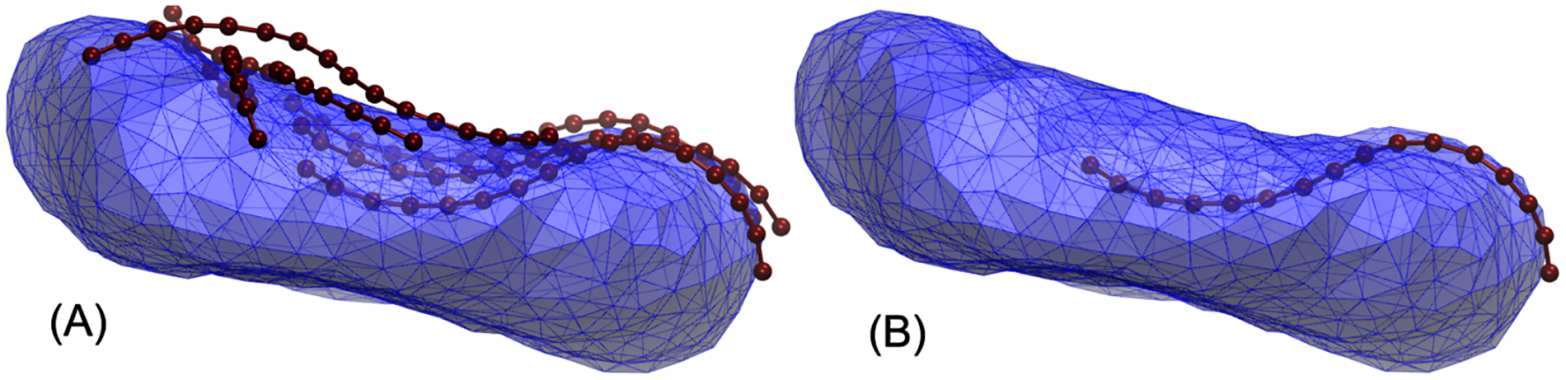
Phagophore formed by flexible Atg17 dimers. (A) Structure from a simulation of phagophore induction by six flexible Atg17 dimers with a stiffness *K*_ang_ = 200 *k*_B_*T*, and a membrane binding strength of *u* = 0.1 (membrane: transparent blue; Atg17 dimers: red). (B) One of the Atg17 dimers shows different curvatures of its two crescents to accommodate better to the phagophore shape.

Interestingly, we found that over a wide range of the chain flexibility the Atg17 dimers are capable of inducing the phagophore with intermediate structures similar to the ones observed for rigid dimers. S4 Video shows phagophore formation for six Atg17 dimers on three fused vesicles in an intermediate binding regime with *u* = 0.1 and for small Atg17 bending stiffness with *K*_ang_ = 200 *k*_B_*T*. The initial membrane structure is identical to that of the intermediate bending regime of rigid Atg17 dimers with *K*_ang_ = 10^3^ *k*_B_*T*, as shown in Fig 5 and S2 Video. Fig 9B shows the final phagophore structure, highlighting one of the six Atg17 dimers shown in Fig 9A. The increased flexibility lets the dimer curve more tightly over the rim of the phagophore shape (right), and flatten at its bottom (left). Overall, we conclude that the phagophore formation is not particularly sensitive to the Atg17 flexibility, as long as membrane binding is tight.

We also performed simulations with different membrane properties including membrane surface tension and bending rigidity. We varied the coefficient *K*_*A*_ of the harmonic potential used to constrain the area from 6 × 10^4^ to 5 × 10^5^ for a vesicle in intermediate binding regime with *u* = 0.1. Upon changing *K*_*A*_, we did not observe any significant change in the success rate of the phagophore formation (see S1 Table), defined as the percentage of vesicles transitioning to the phagophore shape in 35 runs of 10^7^ MC steps each (see Materials and methods). The reason is that the membrane local fluctuations and bulk shape deformations are mainly controlled by the bending rigidity rather than surface tension [29]. In particular, the energy barriers are directly proportional to the membrane bending rigidity. Hence, one expects a more difficult transition towards the phagophore for higher membrane rigidities. Indeed, we found that for more rigid membranes, higher adhesion strengths are required to transform the vesicle. For a bending rigidity of *κ* = 20 *k*_B_*T* the intermediate bending regime occurs at a higher range of binding strengths 0.12 < *u* < 0.2 (see S3 Fig) compared to the lower strengths 0.07 < *u* < 0.14 for *κ* = 10 *k*_B_*T*. This shift is due to higher energy barriers between the tubular and phagophore structures for more rigid membranes. We conclude that stronger binding is required to induce phagophore formation on more rigid membranes.

### Four fused vesicles

Post-fusion membrane structures tend to form tubular vesicles which are separated from the phagophore by two energy barriers *H*_1_ and *H*_2_. The energy barrier *H*_1_ is the critical barrier separating the tube from the disk-like structure. The second energy barrier *H*_2_ between the disk-like vesicle and the phagophore goes away in the event of fusing more than 4 vesicles. However, the first energy barrier *H*_1_ does not vanish even for more fused vesicles as shown in S1 Fig. It is thus the energy barrier *H*_1_ that cannot be crossed spontaneously on relevant timescales.

We confirmed that the phagophore, once formed, is maintained upon fusion of additional vesicles. In principle, the phagophore is energetically favorable when a minimum of there vesicles are fused resulting in a reduced volume *v*_3_ = 0.577. To check whether fusion with additional spherical vesicles maintains the phagophore shape, we fused a spherical vesicle to a cup-shaped phagophore formed previously by fusion of three vesicles. The resulting structure has a reduced volume *v*_4_ = 0.5 corresponding to *n* = 4 fused vesicles. S5 Video shows a movie of the evolution of a vesicle fused with a *v*_3_ phagophore into a phagophore with a smaller reduced volume *v*_4_ = 0.5. As expected the vesicle is rapidly adsorbed, forming a thinner larger phagophore.

We also confirmed that Atg17 dimers can induce tube-to-phagophore transitions for tubes formed by fusion of four instead of three vesicles. The setup contained six Atg17 dimers on initially four fused vesicles, keeping the size of Atg17 dimers and of the fusing vesicles identical with previous simulations with three fused vesicles. S6 Video shows a movie of the vesicle transition towards the phagophore shape through a paddle pathway in an intermediate binding regime with *u* = 0.1.

### Atg17 crescent-surface mutants are defective in autophagy

We sought to test whether contacts between the curved surface of Atg17 and membranes are essential for yeast autophagy. Five mutants were designed in order to make membrane contacts less favorable by replacing surface basic and hydrophobic residues on the Atg17 structure [22] with acidic residues. Surface sites were selected at positions remote from the two known Atg13 binding sites [41, 43], the dimer interface, and contacts with Atg31 [22]. Sites were selected where clusters of exposed basic and/or hydrophobic residues were observed in the *Lachancea thermotolerans* crystal structure, and where these residues were either identically conserved in *Saccharomyces cerevisiae* or replaced with conservative substitutions. The clusters are identified in Fig 4A and Table 1 using *Saccharomyces cerevisiae* numbering. Note that mutants M1 and M2 overlap and represent different probes of a single cluster.

**Table 1:** Mutational analysis.

Lt	Sc	Annotation	Mutant (Sc)	Cluster	Phenotype	WB	Color in Fig 4A
K314	K318	Concave	K318D/Q326D/K329D	M1	20%	Normal	Blue
R325	K329	Concave, partial overlap with M1	K318D/K329D/Y93E/W94E	M2	20%	Slight reduction in expression	Blue and cyan
F16	T16	Convex	K8D/R14D/K249D	M3	60%	Normal	Green
K160	K164	Convex	K164D/K173D/R299D	M4	75%	Normal	Yellow
K93	K92	Convex	K92D/L141D	M5	60%	Doublet	Orange

Lt, *Lachancea thermotolerans*; Sc, *Saccharomyces cerevisiae*; WB, Western blot expression assay. Annotation indicates the location on the Atg17 crescent (see Fig 4A).

Wild-type and mutant *ATG17* alleles were expressed in *Saccharomyces cerevisiae* and assayed for expression by western blotting, and for autophagic activity using the Pho8Δ60 assay [44, 45]. All of the mutants expressed at wild-type or near wild-type levels. A slight reduction in expression was noted for mutant M2, which includes two hydrophobic to Glu changes. The presence of a small amount of an apparent proteolytic fragment was noted for mutant M5. Mutants M1, M3, and M4 were expressed at identical levels to wild-type. All of the mutants ran slightly higher on the gel than wild-type, which was expected because all of the mutations increase the net negative charge.

**Fig 10:**
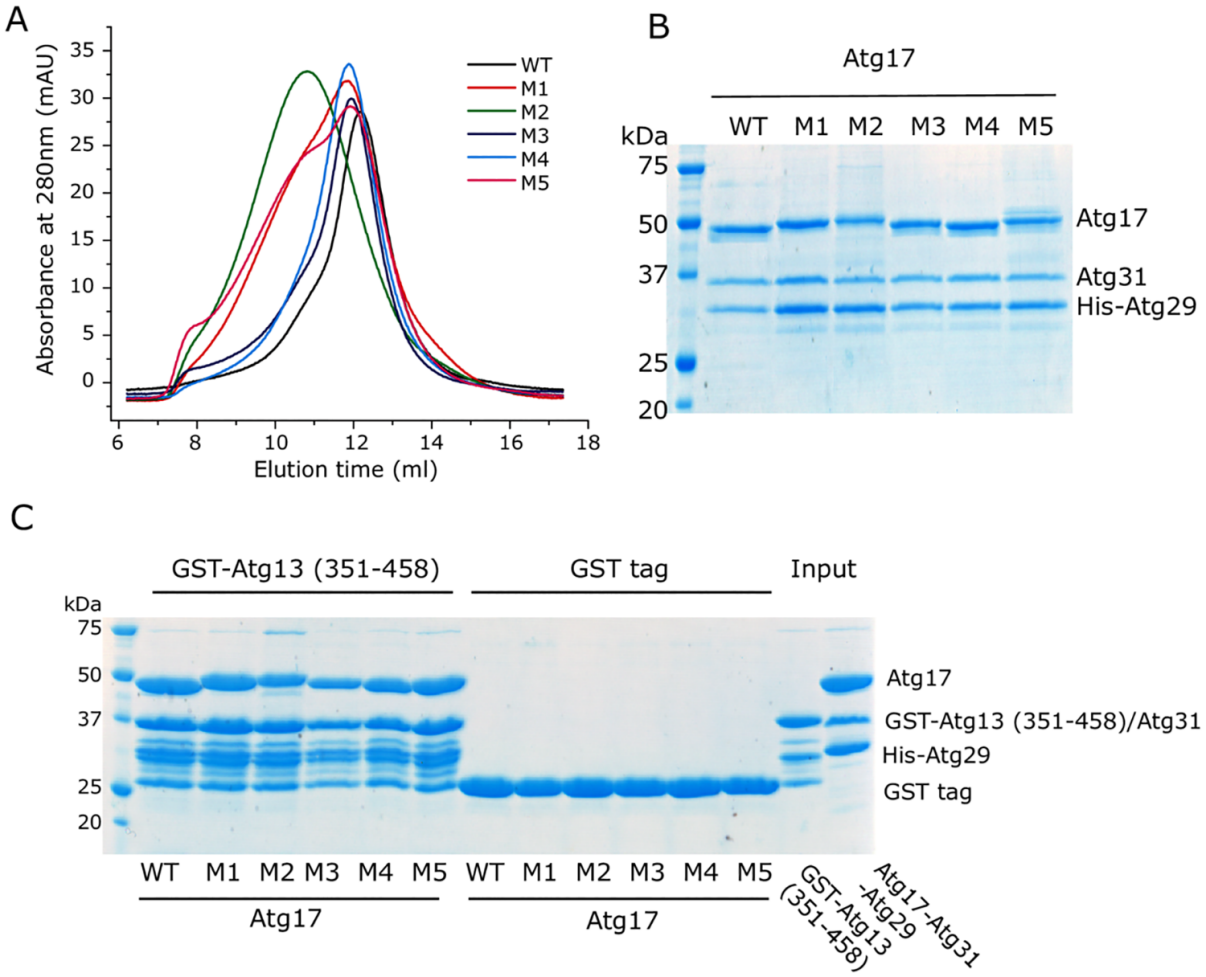
Atg17 mutations do not affect the complex assembly in vitro. The Atg17 mutations were introduced into the Atg17-Atg31-Atg29 construct for E coli expression. (A) Size exclusion chromatography of the recombinant ternary complex showed that the wild-type and mutants eluted in similar positions on a superpose 6 column, suggesting that Atg17 mutations do not interfere with dimerization. (B) SDS-PAGE confirmed that the peak fractions on superpose 6 column contain Atg17, 31, 29. (C) Atg17 mutations do not affect Atg13 binding. GST-Atg13 (351–458) was able to pull down the wild type or mutants of Atg17-Atg31-Atg29 complex with similar affinity.

The data in Fig 10 show that Atg17 mutations do not affect the complex assembly in vitro. In size exclusion chromatography, wild-type and mutant proteins eluted in similar positions. SDS-PAGE and GST-Atg13 pulldown experiments confirmed that Atg17, Atg31, and Atg29 co-assemble, and that Atg13 binding is not affected by the mutations.

**Fig 11:**
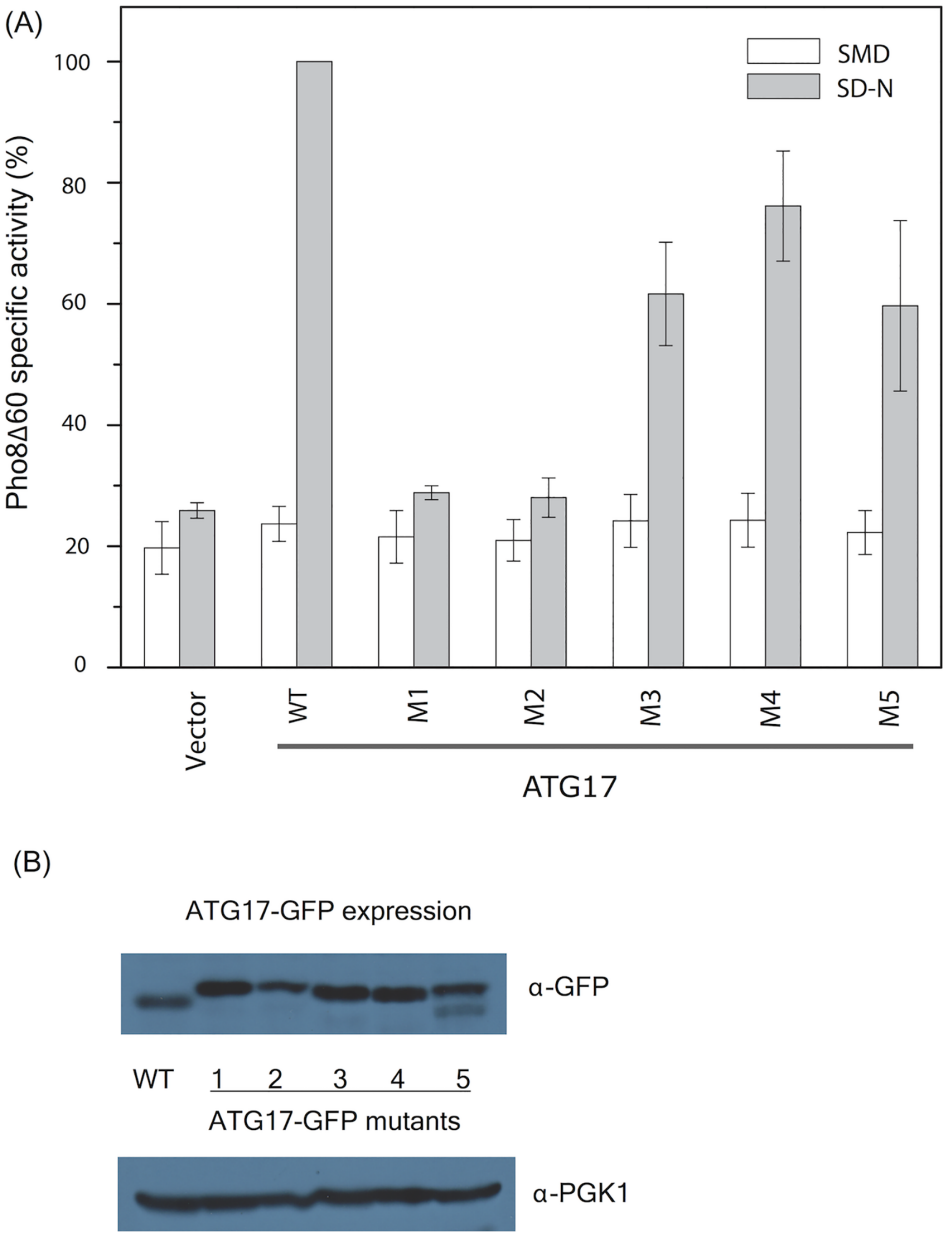
Atg17 crescent surface mutations impair autophagy. (A) Pho8Δ60 assay for monitoring autophagy was performed following growth in rich (white) or nitrogen starvation (grey) media. Error bars represent S.D. of triplicate experiments. (B) The expression of Atg17-GFP was monitored by western blot against GFP. Western blot against 3-phosphoglycerate kinase (PGK1) was used as the loading control for yeast cell extracts.

We then tested the function of the wild type and mutant proteins (Fig 11). The Pho8Δ60 assay measures the vacuolar transport of a phosphatase whose normal vacuolar transport signal has been crippled and is thus dependent on autophagy induction for its transport [44, 45]. This assay is quantitative, making it especially useful for measuring graded responses. We found that mutants M3, M4, and M5 reduce autophagic activity by 25–50% on the basis of this assay. Mutants M1 and M2 completely abrogate Atg17-dependent autophagic activity. M1 and M2 activity is equal to that of the empty vector control (20%; Fig 11).

## Discussion

Forming the phagophore is a crucial, yet poorly understood step in macroautophagy. Recent experiments suggest the fusion of three Atg9 vesicles as the origin of the autophagosomal membrane in yeast [10]. However, the transition from initial fused vesicles towards the phagophore is not trivial. We performed simulations and experiments to study this early phase of phagophore assembly. Our focus was on the induction of the cup-shaped membrane characteristic of the phagophore.

We showed that even though for three vesicles the cup shape is the stable state of the membrane, reaching it requires crossing of two barriers. The second barrier, from disk to cup shapes, is relatively small and disappears altogether for *v* < 0.52 [35], i.e., after four vesicles have fused (with *v*_4_ = 0.5). A similar effect occurs at the “critical” diameter-to-thickness ratio of the disk shapes studied by Knorr et al. [15]. By contrast, the first barrier from tubular shapes typical of the structures after vesicle fusion to disk shapes is high, from 17 to 34 *k*_B_*T* for typical membrane rigidities. This high barrier effectively traps the post-fusion vesicle in a metastable tubular shape. Fusion of additional vesicles does not lower the barrier. Accordingly, in simulations starting from different linear arrangements of different numbers of fusing vesicles, the free membrane adopted a tubular shape, and did not proceed to the phagophore.

We then found that membrane-associated Atg17 dimers establish a viable route to escape from the metastable tubular shapes and proceed to phagophores. A dimer of Atg17 proteins forms the scaffold of the assembled Atg1 complex, adopting a double-crescent shape [22, 23]. In our simulations of membrane remodeling in the presence of Atg17 dimers, this unusual shape turned out to be important (1) for the stabilization of the paddle shapes in the transition from tube to disk shapes; and (2) for the stabilization of the bowl shapes in the transition from disk to cup shapes. Atg17 dimers lower the energy of corresponding transition state structures by binding the membrane, first, with their flat side (Fig 5D) and, second, with the convex side of one Atg17 crescent and the concave side of the other (Figs 4E and 5H).

A possible concern is that the ≈10-nm radius of the Atg17 crescents is somewhat smaller than the 15 to 30-nm Atg9 vesicle radii estimated from imaging experiments in yeast [10]. However, even somewhat larger vesicles should be able to bind to Atg17 dimers from the side, as was suggested also in [23]. Indeed, basic patches on the Atg17 surface are not confined to the convex side (Fig 4A). In this context, we also note that sideways binding is important in some of the membrane remodeling steps seen in the simulations (Figs 5 and 6).

Nevertheless, with the information at hand we cannot rule out other factors. For instance, it is conceivable that the leaflets of the Atg9 vesicles have different lipid and/or protein compositions. Phosphatidylinositol 3-phosphate (PI3P) asymmetry has been demonstrated on autophagosomal membranes [46]. Such asymmetries tend to induce spontaneous membrane curvature *c*_0_, altering the (*c*_1_ + *c*_2_)^2^ term in Eq (1) to (*c*_1_ + *c*_2_ − *c*_0_)^2^. Spontaneous curvature is recognized as a major factor in membrane shape remodeling [47, 48]. It is conceivable that Atg17 acts in concert with other factors driving membrane shape transformations, in particular membrane spontaneous curvature.

Testing the Atg17-mediated phagophore induction by experiment is therefore important. In support of a major role of Atg1, and more specifically of Atg17-membrane interactions, we showed here that mutations of widely distributed surface residues on both the concave and convex faces of Atg17 have significant impact on yeast autophagy. We found that perturbations of likely membrane interaction sites on Atg17, distant from known protein binding sites, have a substantial influence on autophagy induction. In some cases, the mutations led to a complete loss of detectable autophagic activity using the Atg17 pathway.

Our combination of experiments, membrane elasticity theory, and simulations of protein-assisted membrane-remodeling sheds light on the earliest steps in phagophore formation. The fusion of at least three small vesicles [10] emerges as a viable route. However, fusion alone is not sufficient because the resulting vesicle would be kinetically trapped in a tubular shape. The Atg17 dimer core of the Atg1 complex emerges as a key factor. In the simulations, Atg17 dimers facilitate the escape from the trapped state toward the cup-shaped phagophore. The S-shape of Atg17 dimers is ideally suited to scaffold—and thus stabilize—the membrane shapes adopted at the two transition states encountered along the pathway between initial fusion and the phagophore shape.

## Materials and methods

The shape of fluid lipid membranes is governed by the Helfrich bending energy. This elastic energy depends on the bending rigidity *κ* and the membrane geometry, as reflected in the principal curvatures *c*_1_ and *c*_2_ at any given point of a symmetric bilayer with negligible spontaneous curvature. The Helfrich model ignores the asymmetry in the membrane leaflets and assumes negligible membrane thickness compared to the other membrane dimensions. According to the spontaneous curvature model [32], the Helfrich bending energy *E*_b_ is then the integral

$$\begin{array}{c} E_{\text{b}}=\frac{\kappa }{2}\;\oint \;{\left(c_{1}+c_{2}\right)}^{2}dA \end{array}$$ (1)

over the surface area of the vesicle [24].

We assume a typical bending rigidity *κ* = 20 *k*_B_*T* for the lipid membrane in all simulations, where *k*_B_ is the Boltzmann constant and *T* is the temperature. The shape of a vesicle membrane is controlled by the reduced volume $v=6\sqrt{\pi }V/A^{3/2}⩽1$, with *v* = 1 for a spherical vesicle.

For simplicity, we assume that the Atg9 vesicles fusing at the PAS have equal size, and that during the fusion process there is no loss of lipids and exchange of interior content with the outside. The area *A* and the volume *V* of a vesicle membrane are then preserved due to the near incompressibility of membranes and the osmotic balance between the inside and outside of the vesicle [49]. Hence, one can write the reduced volume *v*_*n*_ of a large vesicle formed by fusing *n* equal-size spherical vesicles of volume *V* = 4*πR*^3^/3 and area *A* = 4*πR*^2^ as

$$\begin{array}{c} v_{n}=\frac{6\sqrt{\pi }nV}{{\left(nA\right)}^{3/2}}=\frac{1}{\sqrt{n}} \end{array}$$ (2)

This means that fusion of *n* vesicles lowers the reduced volume by a factor $1/\sqrt{n}$.

### Membrane model

The Helfrich bending energy is discretized using a tessellated mesh generated on the surface of the membrane vesicle. Triangulation of the membrane makes it possible to explore non-axisymmetric shapes [25–31]. The vesicle surface is tessellated into *n*_*t*_ triangles with *n*_*v*_ vertices and *n*_*e*_ edges where *n*_*t*_ − *n*_*e*_ + *n*_*v*_ = 2(1 − *g*) according to Euler’s polyhedron formula. The topological genus of the surface is *g* = 0 for the simply connected vesicles studied here. For the simulations of a free vesicle without Atg17 complexes, we used vesicles with *n*_*v*_ = 2562 vertices, *n*_*e*_ = 7680 edges, and *n*_*t*_ = 5120 triangles. For the simulations with Atg17 complexes, we lowered the resolution to *n*_*v*_ = 642 vertices, *n*_*e*_ = 1920 edges, and *n*_*t*_ = 1280 triangles, making sure that for isolated membranes fine and coarse tessellation produced consistent results.

The Helfrich bending energy in Eq (1) of a tessellated vesicle with *n*_*v*_ vertices is discretized over the vertices and represented by the bending Hamiltonian

$$\begin{array}{c} E_{\text{b}}=2\kappa \;\sum_{\alpha =1}^{n_{v}}\;\frac{{\left(M_{\alpha }\right)}^{2}}{A_{\alpha }} \end{array}$$ (3)

*M*_*α*_ denotes the curvature contribution of vertex *α*, and *A*_*α*_ is the area assigned to the vertex *α*. The mean curvature contribution of the vertex *α* is the sum

$$\begin{array}{c} M_{\alpha }=\frac{1}{4}\;\sum_{ij}\;l_{ij}\phi_{ij} \end{array}$$ (4)

over all edges *ij* that share the vertex *α*. *l*_*ij*_ is the edge length, and *ϕ*_*ij*_ is the angle between two normal vectors of neighbor triangles sharing edge *ij*. The prefactor 1/4 ensures that in the limit of high resolution, the summation approaches the integral of the mean curvature over the surface of the continuous vesicle [25]. The area of a vertex is obtained as the sum

$$\begin{array}{c} A_{\alpha }=\frac{1}{3}\;\sum_{i}\;A_{i} \end{array}$$ (5)

of the areas $A_{i}$ of all neighbor triangles *i* that share the vertex. A prefactor of 1/3 gives the correct area of the whole vesicle as the sum over individual vertex areas, $A=\sum_{\alpha }\;A_{\alpha }=\sum_{i}\;A_{i}$. The average area of a triangle is

$$\begin{array}{c} \bar{A}=\frac{A}{n_{t}} \end{array}$$ (6)

where *A* is the total area. In all tessellations, the lengths of the edges is kept within an interval $\left[l,\sqrt{3}l\right]$, where *l* depends on the average area of triangles $l=\sqrt{4\bar{A}/\sqrt{3}}$.

The volume *V* enclosed by the vesicle is the sum

$$\begin{array}{c} V=\sum_{i=1}^{n_{t}}\;V_{i} \end{array}$$ (7)

of signed volume contributions of triangles

$$\begin{array}{c} V_{i}=\frac{1}{3}\left(n_{i}\cdot R_{i}\right)A_{i} \end{array}$$ (8)

where **n**_*i*_ is the unit normal vector of the triangle *i*, and **R**_*i*_ is the position vector of any point on one of the edges of *i* relative to an arbitrary fixed point. To sample vesicles in Monte Carlo (MC) simulations with reference area *A*_ref_ and volume *V*_ref_, the total energy of the free vesicle is expressed as

$$\begin{array}{c} E=E_{\text{b}}+E_{\text{area}}+E_{\text{vol}} \end{array}$$ (9)

where the stiff harmonic potentials *E*_area_ = *K*_*A*_(1 − *A*/*A*_ref_)^2^ and *E*_vol_ = *K*_*V*_(1 − *V*/*V*_ref_)^2^ are used to preserve the area *A* and volume *V* of the vesicle at reference values *A*_ref_ and *V*_ref_, respectively. In our simulations, we use values of the *K*_*A*_ and *K*_*V*_ coefficients in the order of 10^6^ *k*_B_*T* to restrict area and volume fluctuations of free vesicles to about 0.1%. For the lower resolution vesicles used with Atg17 proteins we used *K*_*A*_ = 2 × 10^5^ *k*_B_*T* and *K*_*V*_ = 5 × 10^5^ *k*_B_*T* to represent physiologically relevant values.

In the simulations, MC moves consist of randomly attempted translations of the vertex positions and flips of edges to ensure the fluidity of the membrane [28, 50]. These MC move attempts are accepted according to the Metropolis criterion, to sample membrane configurations with probabilities proportional to the Boltzmann factor *e*^−*E*/*k*_B_*T*^. The move widths are chosen to achieve an acceptance rate of around 40%.

To find the energy barriers at different reduced volumes we also performed simulated annealing (SA) simulations, in which we minimized the elastic energy of the membrane. In these simulations, the temperature was gradually reduced to values close to zero. To find intermediate membrane conformations we constrained the area difference Δ*a*. The potential energy is then set to

$$\begin{array}{c} E=E_{\text{b}}+E_{\text{area}}+E_{\text{vol}}+E_{\Delta a} \end{array}$$ (10)

where *E*_Δ*a*_ = *K*_Δ*a*_(1 − Δ*a*/Δ*a*_0_)^2^ with *K*_Δ*a*_ = 5 × 10^6^ *k*_B_*T* and Δ*a*_0_ the target area difference. The area difference is defined as the integral of the mean curvature *M* = (*c*_1_ + *c*_2_)/2 over the vesicle surface,

$$\begin{array}{c} \Delta a=\frac{\oint MdA}{2\sqrt{\pi A}} \end{array}$$ (11)

Δ*a* serves as a shape index for the vesicle, where Δ*a* = 1 corresponds to a spherical vesicle [34, 35]. In SA simulations we started from initial spherical vesicles with reduced volume *v* = 1 and linearly decreased the volume to the target reduced volume in 10^6^ MC steps. Then the area difference was linearly varied towards its target value in 10^6^ MC steps. Then the vesicles were equilibrated at the target area difference and reduced volume for 5 × 10^5^ MC steps and cooled down by linearly reducing the temperature to close to zero in 5 × 10^5^ MC steps. For every area difference we performed ten SA simulations and reported the one with minimum elastic energy.

### Coarse-grained Atg17 complex

Protein-assisted remodeling of lipid membranes is of enormous biological relevance. However, describing such complex processes, which occur over length scales exceeding tens of nanometers and time scales exceeding milliseconds, is highly challenging and the focus of ongoing developments [51, 52], with approaches that range from highly coarse-grained descriptions [53] over multiscale simulations [54] to near-analytical models [55–57]. Bradley and Radhakrishnan [57], for instance, elegantly account for the effect of bound proteins on membranes by introducing localized spontaneous curvature fields in a Helfrich-type energy function, with parameters obtained from coarse-grained simulations.

Here, we simulated the interaction of Atg17 dimers with the membrane explicitly. To explore how these interactions affect the evolution of the membrane shape, we incorporated Atg17 dimers into the membrane model at a level of resolution comparable to that of the membrane. Atg17 dimers were represented by 16 beads on a chain. Harmonic bonds, bond angles, and dihedrals were used to preserve the overall shape, as seen in the crystal and solution structures [22, 23]. The S-shaped dimer models were composed of two arcs, each a quarter of a circle with a central angle of 90 degrees (Fig 4A). The circle radius is assumed to be equal to the radius of the three vesicles preceding their fusion. As in the crystal structure, the planes of the two half circles of the S-shaped Atg17 dimer model were tilted by 15 degrees relative to each other.

The total energy in MC simulations of vesicles with associated Atg17 dimers was

$$\begin{array}{c} E=E_{\text{b}}+E_{\text{area}}+E_{\text{vol}}+E_{\text{bd}}+E_{\text{d}} \end{array}$$ (12)

The added terms *E*_bd_ and *E*_d_ are the binding energy between the dimer and the membrane, and the internal potential energy of the Atg17 dimer, respectively. The interactions between the membrane and the dimer are represented as an attractive square-well potential,

$$\begin{array}{c} \varepsilon \left(d\right)=\{\begin{array}{cc} \infty & \;\text{for}\;d⩽d_{0}\\ -UA_{i} & \;\text{for}\;d_{0}<d⩽d_{1}\\ 0 & \;\text{for}\;d>d_{1} \end{array} \end{array}$$ (13)

where *d* is the distance to the closest triangle *i* of the tessellated membrane, $A_{i}$ is the area of this triangle, and *U* is the binding energy per unit area of the vesicle. The distance of closest approach is defined as *d*_0_ = *l*/2, where $l=\sqrt{4\bar{A}/\sqrt{3}}$. If the normal projection of the bead is within a triangle, *d* is the normal distance; otherwise, it is the distance to the closest edge or corner. The binding energy *E*_bd_ is then obtained as the sum of all bound dimer beads

$$\begin{array}{c} E_{\text{bd}}=\sum \;\varepsilon \left(d_{j}\right)=-UA_{\text{bd}} \end{array}$$ (14)

where the sum runs over all bound beads *j* and *A*_bd_ is the total area of the membrane adhered to the dimer beads. We quantify the strength of the interactions in terms of a reduced binding energy coefficient *u* = *UA*_Atg_/(8*πκ*), where *A*_Atg_ is an area defined as the number of beads of a dimer times the average area of each triangle in the tessellation, $A_{\text{Atg}}=16\bar{A}$.

The internal potential energy *E*_d_ of an Atg17 dimer is

$$\begin{array}{c} E_{\text{d}}=\frac{1}{2}K_{\text{bond}}\;\sum \;{\left(\Delta L\right)}^{2}+\frac{1}{2}K_{\text{ang}}\;\sum \;{\left(\Delta \theta \right)}^{2}+\frac{1}{2}K_{\text{dih}}\;\sum \;{\left(\Delta \gamma \right)}^{2} \end{array}$$ (15)

where the first term accounts for bond length fluctuations (Δ*L* = *L* − *L*_0_ where *L* is the distance between two adjacent beads in *l* units and *L*_0_ = 1); the second term accounts for bond angle fluctuations (Δ*θ* = *θ* − *θ*_0_ where *θ* is a bond angle in radians and *θ*_0_ is equal to the reference bond angle corresponding to the dimer structure shown in Fig 4B); and the last term accounts for dihedral angle fluctuations (Δ*γ* = *γ* − *γ*_0_ where *γ* is a dihedral angle in radians and *γ*_0_ is the reference dihedral angle). The sums extend over all distinct bonds, bond angles, and dihedral angles, respectively. Bonding parameters *K*_bond_ = 10^4^ *k*_B_*T*, *K*_ang_ = 10^3^ *k*_B_*T*, and *K*_dih_ = 500 *k*_B_*T* produced a relatively rigid dimer structure. In simulations with flexible Atg17 dimers shown in S4 Video and Fig 9, we used bending stiffnesses in the range 200 *k*_B_*T* ⩽ *K*_ang_ ⩽ 10^3^ *k*_B_*T* to create protein chains with different flexibilities. We also applied repulsive interactions between any pair of protein beads in different dimers, which prevented the beads to come closer than distance *l*. A repulsive potential kept Atg17 beads from crossing the membrane. MC move attempts consisted of random bead displacements in Cartesian space and flipping edges to fulfil membrane fluidity. All MC simulations of vesicles with Atg17 dimers including different binding regimes were run for 10^7^ MC steps. Random simulations of vesicles with Atg17 complexes were repeated 35 times for any set of given parameters. Phagophore formation in 75% of the runs within the given MC time was considered successful.

#### Free energy

To quantify the stability of tubular membrane structures in presence of thermal fluctuations, we assessed the contribution of entropy to the membrane elastic energy by computing the free energy of the vesicle. Potentials of mean force were calculated as a function of the area difference for the typical reduced volume *v* = 0.577 of three fused vesicles. We started from membrane structures obtained in SA simulations at small temperatures and continued simulations at ambient temperature. We performed umbrella sampling for different values of the area difference Δ*a* by adding a harmonic potential energy 0.5*k*(Δ*a* − Δ*a*_0_)^2^ to the system energy *E*. Here, Δ*a*_0_ is the reference area difference of the umbrella sampling window. 6 × 10^4^ data points were sampled from 6 × 10^6^ MC steps per vertex for any umbrella window. Using appropriate values of the harmonic potential strength, *k* = 2 × 10^5^ *k*_B_*T*, we obtained 20 to 30% overlap between adjacent windows for umbrella sampling. We then used the WHAM [36] to combine the collected data and determine the potential of mean force as a function of the area difference Δ*a* at the given reduced volume *v*.

### Molecular dynamics simulations using the coarse-grained MARTINI membrane model

We also performed molecular dynamics (MD) simulations of three fused Atg9 vesicles using the MARTINI force field (version 2) [37], with the GROMACS 4.6.7 MD package [58]. In a first step, a single vesicle membrane was formed from a bilayer patch of 1-palmitoyl-2-oleoyl-sn-glycero-3-phosphocholine (POPC) lipids through a spontaneous bilayer-to-vesicle transition. Three vesicles, each composed of 2048 POPC lipids, were then placed in a 75×45×45 nm^3^ simulation box with approximately 1.1×10^6^ water particles. The vesicles were then fused together by pulling a group of nearby lipids on adjacent vesicles towards each other. We then obtained a pearled tubular structure composed of 6144 POPC lipids after two successive fusions. The system was then simulated for 1.2 *μ*s with a 30 fs time step, a velocity rescaling thermostat set at 310 K, an isotropic pressure coupling set at 1 atm with the Parrinello-Rahman barostat, and periodic boundary conditions [58].

### Yeast autophagy assay

Yeast cells (*Saccharomyces cerevisiae*) were cultured in standard rich medium (YPD) or synthetic dextrose medium (SD, 0.67% yeast nitrogen base with ammonium sulfate, 2% glucose, and appropriate amino acid supplements). Autophagy was induced by shifting to nitrogen starvation medium. TN124 Atg17∷KAN cells were transformed with yCPLAC33 and wild-type and mutant *ATG17*-yCPLAC33 constructs, and grown to mid-log phase. Cells were then switched to nitrogen-starvation media for 3h to induce autophagy and the Pho8Δ60 assay was carried out as previously described [44, 45]. Atg17-GFP expression was characterized by western blotting against GFP with Santa Cruz antibody sc9996.

### Protein expression and purification

*Saccharomyces cerevisiae*, Atg17, Atg29, and Atg31 were subcloned in pST39 vector for recombinant ternary complex expression in E coli [22]. The N-terminus of Atg29 was fused with a His6 tag and a TEV cleavage site. The mutations in Atg17 were introduced into Atg17-Atg31-Atg29 construct by quick-change mutagenesis.

The complex was expressed in E coli BL21 (DE3) cell and induced at 0.5 mM IPTG at 37°C for 3 hours. The cells were lysed by sonication in a buffer containing 50 mM Tris pH 8.0, 300 mM NaCl, 20 mM Imidazole, 3 mM *β*-mercaptoethanol (BME), and 1 mM PMSF. The clarified lysate was purified on Ni-NTA resin (Qiagen, Germantown, MD). The elute was further loaded onto a superpose 6 10/300GL column (GE healthcare, Pittsburgh, PA) in a buffer containing 20 mM Tris at pH 8, 200 mM NaCl, and 0.1 mM TCEP.

*Saccharomyces cerevisiae* Atg13 fragment (351–458) was subcloned into a pGST2 vector [59], fused with an N-terminal GST tag and a TEV cleavage site. The clarified lysate was purified on Glutathione Sepharose 4B (GE healthcare), then loaded onto a HiLoad superdex 200 16/60 column (GE healthcare) in the same buffer.

### GST pull-down assay

20 *μ*g of recombinant GST proteins immobilized to 30 *μ*L Glutathione Sepharose 4B resin were incubated with the wild type or mutants of Atg17-Atg31-Atg29 complex (10 *μ*M) at 4°C for 1 hour in the buffer of 20 mM Tris at pH 8, 150 mM NaCl, 0.1 mM TCEP. The beads were washed 3 times, mixed with 35 *μ*L of 2x LDS buffer and boiled for 3 min. 10 *μ*L of each sample was subjected to SDS/PAGE gel.

## Supporting information

S1 Video Coarse-grained MD equilibration of three fused POPC vesicles. Coarse-grained molecular dynamics simulation of three initially fused vesicles with *v* = 0.577. The initial pearled tube relaxed into the metastable cylindrical tube and remained stable during 1.2 *μs*. The tubular vesicle contains 6144 lipids. The 1.1 × 10^6^ water particles are not shown.
(AVI)

S2 Video Atg17-mediated phagophore induction following the paddle pathway. The cooperative interaction of the S-shaped Atg17 dimers bound to the membrane transformed the initial structure, corresponding to the state after fusion of three vesicles, to the final phagophore cup shape. In the intermediate binding regime, the pathway is characterized by an intermediate paddle structure.
(AVI)

S3 Video Atg17-mediated phagophore induction following the starfish pathway. The strong binding of Atg17 dimers to the membrane transformed the membrane from an initial structure, corresponding to the state after fusion of three vesicles, through intermediate starfish structures to a bowl shape. The subsequent transition from the bowl to the phagophore is similar to the one in the paddle pathway in S2 Video, and is therefore not shown.
(AVI)

S4 Video Atg17-mediated phagophore induction by flexible Atg17 dimers. Phagophore formation is induced along the paddle pathway by Atg17 dimers with small bending stiffness *K*_ang_ = 200 *k*_B_*T* in an intermediate Atg17-binding regime *u* = 0.1. The initial structure was identical to that of the simulation in the intermediate binding regime with rigid Atg17 dimers, *K*_ang_ = 10^3^ *k*_B_*T*, as shown in S2 Video and Fig 5. In the final frames of the movie, the phagophore is rotated around the *x* axis to visualize its shape.
(AVI)

S5 Video Fusion of a single vesicle to a phagophore formed from three vesicles. A phagophore already formed by fusing three vesicles with a reduced volume *v*_3_ = 0.577 is fused to a fourth vesicle. The vesicle is rapidly adsorbed into the phagophore and a new thinner phagophore with larger internal space and a smaller reduced volume *v*_4_ = 0.5 is formed. Adding more vesicles would similarly lead to growing phagophores with larger internal space and smaller reduced volumes.
(AVI)

S6 Video Atg17-mediated phagophore induction from initially four fused vesicles. The phagophore is formed through a paddle pathway with six rigid Atg17 dimers on initially four fused vesicles in an intermediate binding regime *u* = 0.1. The size of the Atg17 dimers and initial vesicles are identical with that of three fused vesicles, shown in S2 Video and Fig 5. In the final frames, the phagophore is rotated around the *x* axis to visualize its shape.
(AVI)

S1 FigBending energy curves for different numbers of fused vesicles.Shape branches for different reduced volumes $v_{n}=1/\sqrt{n}$ corresponding to different numbers *n* of the fused vesicles, as indicated for each branch (with *n* in parentheses). The energy barriers between tube-shaped and disk-shaped vesicles persist even as *n* is increased and *v*_*n*_ shrinks. The energetics of the membrane tube-to-sheet transition in the regime of narrow tubes is explored in more detail in [14].(TIFF)

S2 FigVesicle shape relaxation dynamics in molecular dynamics simulations.Autocorrelation function (blue line) of the radius of gyration *R*_*g*_ of the tubular vesicle in Fig 3C from molecular dynamics simulations. The red line is a biexponential fit, $we^{-t/t_{1}}+\left(1-w\right)e^{-t/t_{2}}$ with *w* = 0.5, *t*_1_ = 0.003 *μ*s and *t*_2_ = 0.051 *μ*s.(TIFF)

S3 Fig Phagophore formation with rigid membranes. The phagophore shape of the vesicle (blue) was induced by six Atg17 complexes (red) in an intermediate binding regime with *u* = 0.15 for a rigid membrane with *κ* = 20 *k*_B_*T*. Stiffening *κ* from 10 to 20 *k*_B_*T* shifts the intermediate regime from 0.07 < *u* < 0.14 to 0.12 < *u* < 0.2.
(TIFF)

S1 Table Effect of area constraint *K*_*A*_ on success rate of phagophore induction for Atg17 binding strength *u* = 0.1. The success rate is the percentage of simulation runs in which the Atg17-bound vesicles transitioned from a tubular to a phagophore shape. For each *K*_*A*_, 35 runs of 10^7^ MC steps were performed.
(PDF)
