# Supplementary figures and images for: Scaffolding the cup-shaped double membrane in autophagy
Source: PLoS Comput Biol. 2017 Oct 24;13(10):e1005817. doi: 10.1371/journal.pcbi.1005817 (PMC5669500; doi:10.1371/journal.pcbi.1005817)

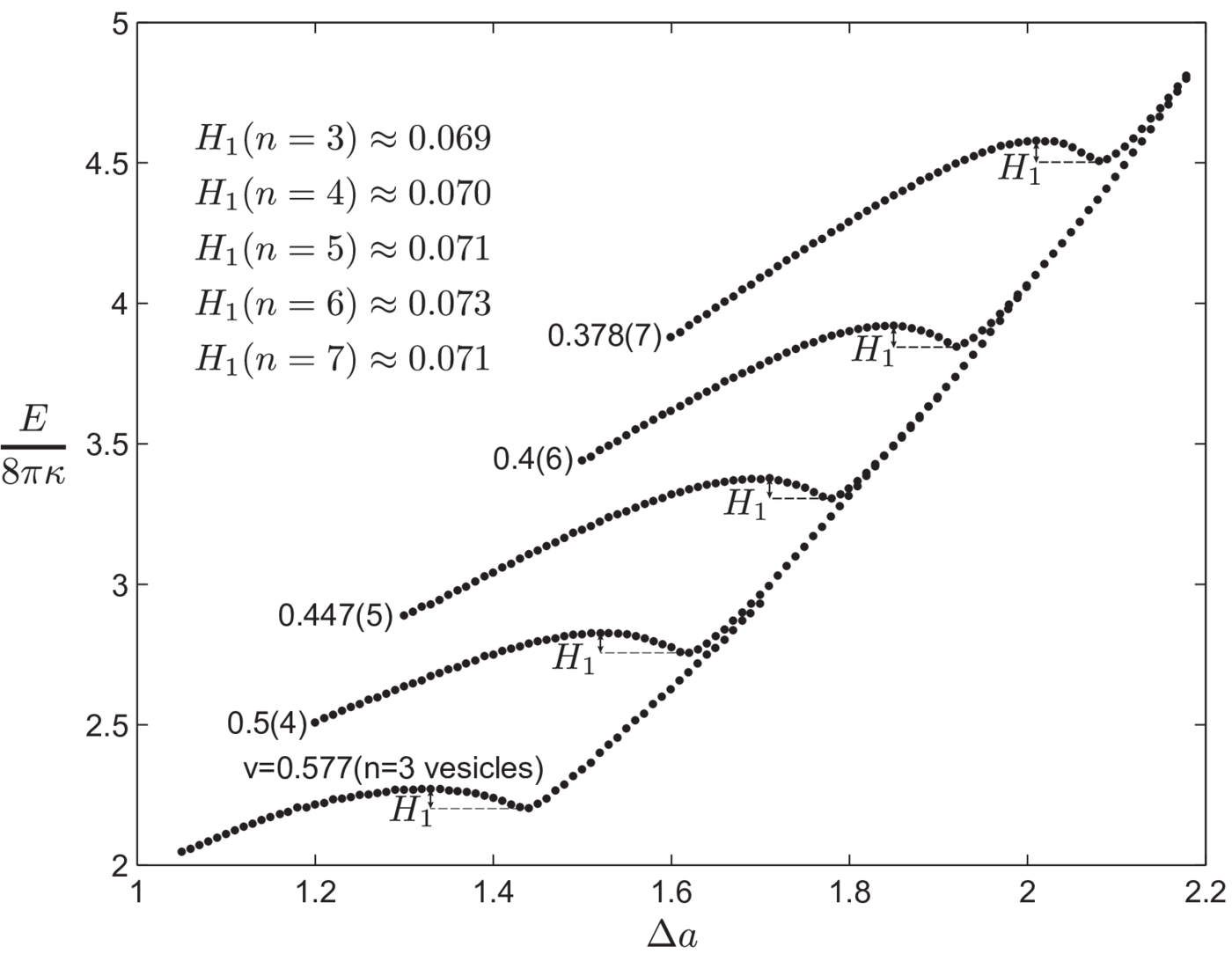

Supplement: S1 Fig — Shape branches for different reduced volumes vn=1/n corresponding to different numbers n of the fused vesicles, as indicated for each branch (with n in parentheses). The energy barriers between tube-shaped and disk-shaped vesicles persist even as n is increased and vn shrinks. The energetics of the membrane tube-to-sheet transition in the regime of narrow tubes is explored in more detail in [14]. (TIFF) [file pcbi.1005817.s007.tiff]

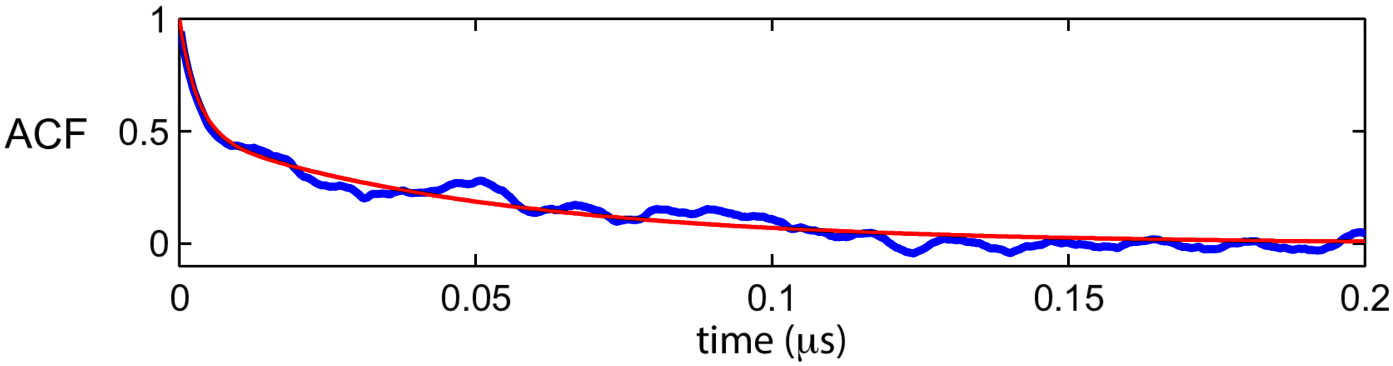

Supplement: S2 Fig — Autocorrelation function (blue line) of the radius of gyration Rg of the tubular vesicle in Fig 3C from molecular dynamics simulations. The red line is a biexponential fit, we-t/t1+(1-w)e-t/t2 with w = 0.5, t1 = 0.003 μs and t2 = 0.051 μs. (TIFF) [file pcbi.1005817.s008.tiff]

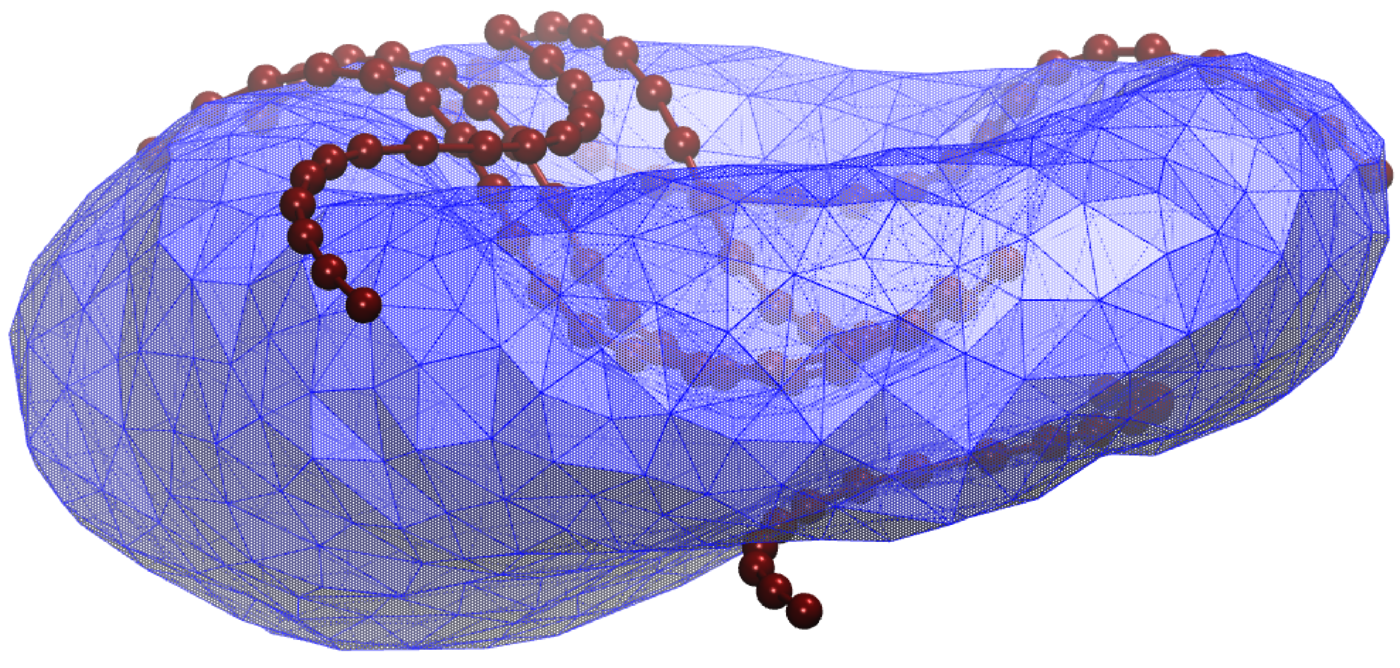

Supplement: S3 Fig — The phagophore shape of the vesicle (blue) was induced by six Atg17 complexes (red) in an intermediate binding regime with u = 0.15 for a rigid membrane with κ = 20 kBT. Stiffening κ from 10 to 20 kBT shifts the intermediate regime from 0.07 < u < 0.14 to 0.12 < u < 0.2. (TIFF) [file pcbi.1005817.s009.tiff]
