# Supplementary material for: Scaffolding the cup-shaped double membrane in autophagy
Source: PLoS Comput Biol. 2017 Oct 24;13(10):e1005817. doi: 10.1371/journal.pcbi.1005817 (PMC5669500; doi:10.1371/journal.pcbi.1005817)
Supplement: S1 Table — The success rate is the percentage of simulation runs in which the Atg17-bound vesicles transitioned from a tubular to a phagophore shape. For each KA, 35 runs of 107 MC steps were performed. (PDF) [file pcbi.1005817.s010.pdf]

$K_A/(10^5\, k_{\mathrm{B}}T)$	0.6	1	1.4	1.8	2.2	2.6	3	3.4	3.8	4.2	4.6	5
Success [%]	82	85	82	80	82	77	82	77	80	77	77	85
